# Neurodegenerative Diseases: Unraveling the Heterogeneity of Astrocytes

**DOI:** 10.3390/cells13110921

**Published:** 2024-05-27

**Authors:** Alberto Santiago-Balmaseda, Annai Aguirre-Orozco, Irais E. Valenzuela-Arzeta, Marcos M. Villegas-Rojas, Isaac Pérez-Segura, Natalie Jiménez-Barrios, Ernesto Hurtado-Robles, Luis Daniel Rodríguez-Hernández, Erick R. Rivera-German, Magdalena Guerra-Crespo, Daniel Martinez-Fong, Carlos Ledesma-Alonso, Sofía Diaz-Cintra, Luis O. Soto-Rojas

**Affiliations:** 1 Laboratorio de Patogénesis Molecular, Laboratorio 4 Edificio A4, Carrera Médico Cirujano, Facultad de Estudios Superiores Iztacala, Universidad Nacional Autónoma de México, Mexico City 54090, Mexico; albertosantiago@comunidad.unam.mx (A.S.-B.); annai.orozco@cinvestav.mx (A.A.-O.); marcusvillegas3@gmail.com (M.M.V.-R.); dr.isaacps91@gmail.com (I.P.-S.); ernestohurtador23@gmail.com (E.H.-R.); luis.d.rodriguez.h@comunidad.unam.mx (L.D.R.-H.); richar_rich@live.com (E.R.R.-G.); 2 Departamento de Fisiología, Biofísica y Neurociencias, Centro de Investigación y de Estudios Avanzados del Instituto Politécnico Nacional, Mexico City 07360, Mexico; irais.valenzuela@cinvestav.mx (I.E.V.-A.); natalie.jimenez@cinvestav.mx (N.J.-B.); daniel.martinezfong@cinvestav.mx (D.M.-F.); 3 Sección de Estudios de Posgrado e Investigación, Escuela Superior de Medicina, Instituto Politécnico Nacional, Ciudad de Mexico 11340, Mexico; 4 Laboratorio de Medicina Regenerativa, Departamento de Fisiología, Facultad de Medicina, Universidad Nacional Autónoma de Mexico, Mexico City 04510, Mexico; mguerra@facmed.unam.mx; 5 Departamento de Neurobiología del Desarrollo y Neurofisiología, Instituto de Neurobiología, Universidad Nacional Autónoma de Mexico, Querétaro 76230, Mexico; neurovet.cla@gmail.com

**Keywords:** neurotoxic and neuroprotective astrocytes, Alzheimer’s disease, Parkinson’s disease, Huntington’s disease, multiple sclerosis

## Abstract

The astrocyte population, around 50% of human brain cells, plays a crucial role in maintaining the overall health and functionality of the central nervous system (CNS). Astrocytes are vital in orchestrating neuronal development by releasing synaptogenic molecules and eliminating excessive synapses. They also modulate neuronal excitability and contribute to CNS homeostasis, promoting neuronal survival by clearance of neurotransmitters, transporting metabolites, and secreting trophic factors. Astrocytes are highly heterogeneous and respond to CNS injuries and diseases through a process known as reactive astrogliosis, which can contribute to both inflammation and its resolution. Recent evidence has revealed remarkable alterations in astrocyte transcriptomes in response to several diseases, identifying at least two distinct phenotypes called A1 or neurotoxic and A2 or neuroprotective astrocytes. However, due to the vast heterogeneity of these cells, it is limited to classify them into only two phenotypes. This review explores the various physiological and pathophysiological roles, potential markers, and pathways that might be activated in different astrocytic phenotypes. Furthermore, we discuss the astrocyte heterogeneity in the main neurodegenerative diseases and identify potential therapeutic strategies. Understanding the underlying mechanisms in the differentiation and imbalance of the astrocytic population will allow the identification of specific biomarkers and timely therapeutic approaches in various neurodegenerative diseases.

## 1. Introduction

The brain, the most intricate vertebrate organ, comprises a complex array of cell types that collaborate harmoniously to regulate every aspect of its biology. These cell types have evolved to perform distinct but interconnected physiological actions to attain the correct brain functioning [1].

Glial cells are the dominant cell type in the central nervous system (CNS) and play a crucial role in enabling the brain to function as both an organ and a computational structure. These cells constitute around 60% of the brain mass and are classified as microglia and macroglia [1]. Microglia comprise approximately 10% of CNS glial cells and participate in brain development, maintenance of neuronal networks, and the professional immune response of the CNS [2]. In the latter function, microglia act as vigilant cells of the extracellular environment that rapidly respond to minor changes or noxious insults to neighboring neural cells or non-CNS immune cells, recruiting astrocytes and other cells to control the damage [3]. Within the CNS, macroglia can be subdivided into oligodendrocytes and astrocytes. Astrocytes represent at least 50% of human brain cells and perform a great variety of activities. They have long been considered the structural support of neurons and the blood–brain barrier (BBB). They also actively participate in neuronal excitability and neurotransmission, maintaining ion balance, buffering neurotransmitters, and regulating brain metabolism. Recently, astrocytes have been found to be involved in neuroplasticity, facilitating synaptogenesis and secreting neuroactive substances, and in the professional immune response of the CNS [4]. Additionally, astroglial cells have important roles in the glymphatic system, a network of specialized perivascular channels that efficiently remove soluble proteins, metabolites, and waste from the CNS [5].

In the early 1900s, Ramón y Cajal meticulously documented the morphological differences of human astrocytes, hinting at the existence of multiple subtypes of astrocytes [6]. Since then, increasing numbers of works have tried to correlate the numerous physiologic roles of astrocytes with different phenotypes. This correlation is evident in the role of astrocytes in neuroinflammation. In this context, attempts have been made to classify astrocytes as neurotoxic or neuroprotective according to respective phenotypes that comprise various molecular changes in response to different situations.

In 2012, Zamanian et al. [7] identified two distinct phenotypes or states of reactive astrocytes through transcriptome analysis in brain ischemia and neuroinflammation models. They associated one phenotype with detrimental effects and the other with protective effects. The specific astrocytic phenotype depended on the induced injury, with a significant difference of at least 50% in gene expression. In 2017, Liddelow et al. [8] demonstrated that lipopolysaccharide (LPS)-activated microglia induce a neurotoxic astrocyte phenotype since then referred to as “A1” through the secretion of three cytokines: interleukin-1 alpha (IL-1α), tumor necrosis factor-alpha (TNFα), and complement component subunit 1q (C1q). Recently, it has been shown that activated endothelial cells (LPS-treated) can also induce astrocytic reactivity similar to that of the “A1” phenotype, but with different molecular marks than that induced by microglia and maintaining their phagocytic capacity, releasing cytokines with a profile different from that of microglia [9]. The relevance of A1 reactive astrocytes in neurodegenerative diseases relies on the findings that dysfunctional astrocytes can cause neuronal death and contribute to neural disturbances [4].

Conversely, the “A2” astrocytic phenotype promotes neuroprotection. In response to CNS injury or neuroinflammation, A2 astrocytes release anti-inflammatory molecules, such as cytokines, chemokines, and neurotrophic factors [10]. Considerable progress has been made in differentiating between those phenotypes or states of astrocytes based on their morphology, proliferation profile, molecular expression, functions, and interactions with other cells [11]. On this basis, a consensus has been reached that reactive astrocytes can assume multiple states depending on the context of their appearance, with only a fraction of common changes between different states and without polarizing into simple binary phenotypes [12]. Hence, the research on reactive astrocytes should include analyses of multiple molecular and morphofunctional parameters.

Therefore, this review delves into astrocyte diversity, analyzing molecular, cellular, and physiological characteristics. Since the extensive scientific evidence, this review also deals with the studies on A1 and A2 astrocytes in neurodegenerative diseases. Finally, this review also analyzes possible therapeutic strategies that modulate astrocytic reactivity in these neurological disorders, considering that no drugs are currently used in clinical practice to target specific astrocyte phenotypes.

## 2. Intracellular Signaling Pathways Regulating Astrocytic Phenotypes

This review proposes that activating intracellular signaling pathways triggers astrocyte reactivity in neurological diseases, whereby astrocytes adopt distinct structural and functional states [13,14]. However, the exact mechanisms behind this process are still unclear. Understanding these mechanisms is crucial for developing effective treatments for neurodegenerative diseases. In this context, this review discusses the intracellular signaling pathways well identified so far in astrocytic polarization. These pathways are Toll-like receptors (TLRs), nuclear factor kappa-B (NF-κB), Ras homolog family member A/Rho kinase (RhoA/ROCK), Janus kinase/signal transducer and activator of transcription 3 (JAK/STAT3) pathway, and phosphoinositide 3-kinase (PI3K)/AKT pathway [4].

### 2.1. Signaling Pathways in Astrocytes Associated with Neurotoxic Mediators

Astrocytes respond differentially to CNS stimuli that activate different receptors and the respective signaling intracellular pathways, whose outcome can be a harmful or protective effect. 

**Figure 1:**
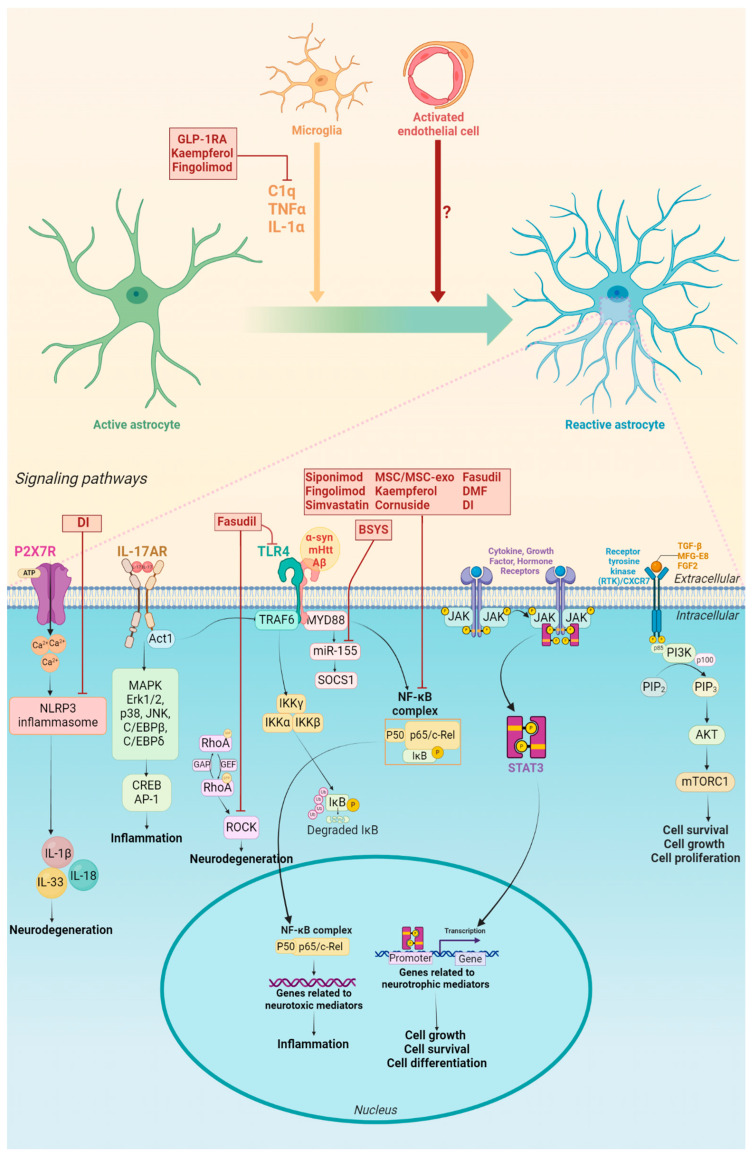
Key pathways of astrocytes in neurodegeneration and potential treatments. Active astrocyte refers to physiological roles. Abbreviations: α-Syn: alpha-synuclein; Aβ: amyloid beta; AKT: serine/threonine-protein kinases; AP-1: activator protein-1; BSYS: Bu Shen Yi Sui capsule; C/EBPB: CCAAT enhancer binding protein beta; CEBPD: CCAAT enhancer binding protein delta; C1q: complement component subunit 1q; CREB: cAMP response element-binding protein; c-Rel: proto-oncogene c-Rel; CXCR7: chemokine receptor 7; DI: dimethyl itaconate; DMF: dimethyl fumarate; Erk 1/2: extracellular signal-regulated kinases 1/2; GAP: GTPase-activating protein; GEF: guanine nucleotide exchange factor; GLP-1RA: glucagon-like peptide-1 receptor agonists; FGF2: fibroblast growth factor 2; IκB: inhibitor of NF-κB; IKK (α, β, γ): inhibitor of nuclear factor-κB (IκB) kinase α, β, γ; IL-1α: Interleukin-1 alpha; IL-1β: interleukin-1 beta; IL-18: interleukin-18; IL-33: interleukin-33; IL-17AR: interleukin-17A receptor; JAK2/STAT3: Janus kinase 2/signal transducer and activator of transcription 3; JNK: jun n-terminal kinase; MAPK: mitogen-activated protein kinase; MFG-E8: milk fat globule epidermal growth factor 8; mHtt: mutant huntingtin; MSC: mesenchymal stem cell; MSC-exo: mesenchymal stem cell exosomes; mTORC1: mTOR complex 1; MYD88: myeloid differentiation primary response protein 88; NF-κB: nuclear factor kappa-B; NLRP3: nod-like receptor protein 3; PI3K: phosphoinositide 3-kinase; PIP2: phosphatidylinositol 4,5-bisphosphate; PIP3: phosphatidylinositol 3,4,5-trisphosphate; P2X7R: P2X7 receptor; p38: p38 protein; p50: p50 protein; p65: p65 protein; RhoA: Ras homolog family member A; ROCK: Rho kinase; SOCS1: suppressor of cytokine signaling 1; TGF-β: transforming growth factor beta; TLR4: Toll-like receptor 4; TNFα: tumor necrosis factor-alpha; TRAF6: tumor necrosis factor receptor-associated factor 6. The red boxes indicate the possible therapeutic targets that modulate the reactivity of A1 and A2 astrocytes. Created with BioRender.com.

TLR activation is known to lead to the conversion of active astrocytes into reactive astrocytes in neurodegenerative diseases. Thus, TLR4 stimulation in astrocytes favors the appearance of the A1 phenotype [8,15]. This receptor is also expressed in the cell membranes of microglia in the CNS, whose activation causes microglial activation and the consequent secretion of IL-1α, TNFα, and C1q, which are required for A1 astrocyte differentiation (Figure 1) [8].

Several studies have analyzed the role of TLRs in modulating astrocyte function in vitro using pathogen-derived components, endogenous TLR activators, synthetic agonists, and antagonists as ligands. The common outcome is that the expression or release profile of cytokines and chemokines depends on the engaged TLR and the ligand type for the TLR [16,17,18].

The transcription factor *NF-κB* pathway, where several intracellular signaling pathways converge, including TLR activation, has been extensively studied to discriminate the role of astrocytes in neuroinflammation and immune response. Inactive NF-κB is found in the cytoplasm, bound to the protein inhibitor NF-κB alpha (IκBα). Once activated, NF-κB translocates into the cell nucleus to activate the expression of genes coding for pro-inflammatory cytokines, thus intensifying and amplifying the inflammatory response [19]. Some studies propose that the NF-κB signaling pathway preferentially mediates the modulation of astrocyte population in neurological diseases. Such a proposal emerges from findings in astrocytes that neurons and reactive microglia release factors that activate the IκB kinase complex (IKK), resulting in IκBα phosphorylation and degradation. Subsequently, p50 and p65, two NF-κB subunits, translocate to the nucleus and activate the transcription of genes related to neuroinflammation in astrocytes (Figure 1) [20,21]. In a mouse model of depression induced by mild chronic stress, it was found that the NF-κB pathway in microglia activates the Nod-like receptor protein 3 (NLRP3) inflammasome, which finally leads to caspase-1 activation. Then, active caspase-1 triggers the release of TNFα, IL-1α, and C1q, eliciting the reactivity of neurotoxic A1 astrocytes [22]. Likewise, the activation of NLRP3 and induction of A1 astrocytes was also demonstrated in primary cultures of murine astrocytes stimulated with lipopolysaccharide (LPS) [23]. NLRP3 can also be activated by P2X purinoceptor 7 (P2X7R) stimulation, which is expressed in microglia and astrocytes [24]. P2X7R stimulation is known to induce the release of bioactive molecules, including pro-inflammatory cytokines, chemokines, proteases, reactive oxygen species (ROS), nitrogen molecules, and excitotoxic glutamate, all capable of causing neurodegeneration [24]. P2X7R is also involved in the ATP-induced membrane pores that allow an excessive influx of Ca^2+^ and depletion of intracellular ions and metabolites, ultimately leading to the lysis of antigen-presenting cells as microglia (Figure 1). This mechanism has given rise to the hypothesis that P2X7R is a “death/suicide” receptor [25,26].

Another signaling pathway whose activation has been implicated in the pathological role of astrocytes is the RhoA/ROCK pathway (Figure 1) [27,28,29]. RhoA and its downstream effector ROCK are ubiquitously expressed in the CNS, including astrocytes and neurons [27]. The RhoA/ROCK signaling pathway may induce microglia and astrocyte activation and increase the expression of nitric oxide synthase and TNFα, which cause neurodegeneration. In addition, recovery from neurodegeneration is blocked because ROCK inhibits cell survival and axon growth [29]. 

Astrocytes, like other neuronal cells, also express the interleukin-17A receptor (IL-17AR), which is a heterodimeric complex (Figure 1) [30,31]. It has been suggested that IL-17AR can enhance glutamate excitotoxicity by reducing the ability of astrocytes to absorb and metabolize glutamate [32]. Moreover, IL-17AR expression is increased in astrocytes in the CNS of mice with experimental autoimmune encephalomyelitis, thus reinforcing the role of this receptor in neurodegeneration [30]. 

In summary, some essential signaling pathways activated by different insults have been identified that can mediate the astrocyte compromise for a harmful reactive state during brain injuries. However, a more profound understanding of the mechanisms determining the phenotype of reactive astrocytes in neuroinflammation, and neurodegeneration is needed to develop effective treatments against these pathologies.

### 2.2. Signaling Pathways of Astrocytes Associated with Neurotrophic Mediators

Recently, research has focused on discovering strategies that activate signaling pathways to induce homeostatic astrocytes and neurotoxic astrocytes toward neuroprotective astrocytes, aiming to implement new treatments against neurodegeneration. However, the mechanism by which they are generated needs to be better understood. Despite this limitation, signaling pathways that could promote the transition to neuroprotective astrocytes have been proposed.

The JAK/STAT3 signaling pathway activated by receptors to cytokine, growth factors, or hormones is thought to be linked to the induction of neuroprotective reactive astrocytes (Figure 1) [33]. Upon activation of the JAK/STAT3 pathway, JAK phosphorylates tyrosine residue (Tyr705) in the C-terminal domain of STAT3, thus causing STAT3 dimerization and translocation to the nucleus to initiate the transcription of target genes [33,34]. In astrocytes, STAT3 activation can be triggered by IL-6 receptor activation, often induced by the release of IL-6 mediated by NF-κB [35]. Consequently, IL-6, via STAT3 phosphorylation, modulates the production of IL-10 [36], an anti-inflammatory cytokine known to promote neuronal survival [37]. This pathway has also been associated with the infiltration of leukocytes, maintenance of myelin, generation of anti-inflammatory cytokines, and other physiological processes such as cell growth, survival, or differentiation [34]. Moreover, it has been proposed that the JAK/STAT3 pathway promotes neurogenesis and neuronal protection in the CNS [38,39]. In a murine model of ischemic brain injury with genetic estradiol depletion, researchers observed that the JAK/STAT3 signaling pathway is downregulated. This downregulation was associated with attenuated reactive astrogliosis, lower transcription of the “A2” panel of reactive astrocyte genes in the hippocampus, microglial activation, enhanced neuronal damage, and cognitive deficits. These effects were reversed after the signaling pathway was recovered through exogenous estradiol administration [40].

The PI3K/AKT pathway (Figure 1) has also been related to the neuroprotective role of astrocytes [41]. This pathway is activated when a ligand, which may be a growth factor or hormone, binds to a tyrosine kinase receptor located on the plasma membrane. Upon ligand–receptor binding, phosphorylation is produced on tyrosine residues in the intracellular domain of the receptor, causing its dimerization. Subsequently, the regulatory subunit p85 of PI3K binds to the phosphorylated tyrosine residues of the receptor to recruit the catalytic subunit p100, forming the active PI3K enzyme. PI3K phosphorylates phosphoinositides, producing three lipid products bound to the cell membrane: PIP, PIP2, and PIP3. Among these lipid products, PIP3 binds to the Pleckstrin homology domain of the serine/threonine protein kinase AKT, leading to its translocation to the membrane and complete activation. Through activating mTORC1, active AKT triggers downstream responses in the cell, such as cell growth, proliferation, and survival [42]. 

Some studies suggest that the activation of the PI3K/AKT pathway promotes astrocytic reactivity towards a neuroprotective phenotype while excluding the neurotoxic phenotype [41,43]. This pathway can be activated by transforming growth factor beta (TGF-β) [44] released by M2 macrophages [41,45] and neuroprotective astrocytes. It is even thought that TGF-β secreted by A2 astrocytes is essential in neuroprotection since it could act on other immune cells as macrophages or infiltrating T lymphocytes (Figure 1) [46,47]. In 2023, in co-cultures of M2 macrophages with spinal cord astrocytes, Pang et al. demonstrated that M2 macrophages stimulate astrocytes to secrete IL-10, IL-13, and TGF-β, as well as facilitate S100A10 marker expression in astrocytes. The authors also showed that antagonists of the TGF-β receptor and inhibitors of PI3K reverse the secretion of anti-inflammatory cytokines and TGF-β [41]. In a murine brain injury model, Divolis et al. (2019) showed that TGF-β administration increased glial fibrillary acidic protein (GFAP) and S100A10 mRNA levels in astrocytes and decreased C3 mRNA [48]. These results suggest that TGF-β may activate signaling pathways, such as PI3K/AKT, which would trigger reactive astrocytes to transform into neuroprotective astrocytes.

Furthermore, the PI3K/AKT pathway can also be activated by milk fat globule epidermal growth factor 8 (MFG-E8) (Figure 1). Xu et al. (2018) induced the activation of A1 astrocytes in vitro by adding a conditioned medium of microglia activated by β-amyloid (Aβ42). They found that MFG-E8 inhibited the expression of the A1 astrocyte marker C3 in response to the conditioned medium while increasing the expression of TGF-β, a marker of neuroprotective astrocytes. In contrast, TGF-β expression in A2 astrocytes decreased in the presence of a PI3K inhibitor. Therefore, the authors concluded that MFG-E8 plays a regulatory role in A1 and A2 astrocytic states through the positive regulation of the PI3K-AKT pathway [46]. It has also been reported that fibroblast growth factor 2 (FGF2) can activate the PI3K/AKT pathway (Figure 1). Feng et al. (2023) evaluated the effect of administering 2,3,5,6-tetramethylpyrazine (TMP), an active component of Ligusticum chuanxiong Hort, on astrocytic reactivity through a model of permanent occlusion of the middle cerebral artery. The aim was to improve neurovascular restoration in subacute ischemic stroke. The authors determined that TMP decreased the number of C3-positive A1 astrocytes and, conversely, increased the number of S100A10-positive A2 astrocytes and FGF2-positive astrocytes. TMP also enhanced the levels of PI3K p85/p55 and AKT and upregulated the expression of FGF2. Therefore, it was concluded that TMP caused the release of FGF2, which activated the PI3K/AKT pathway, thereby converting A1 astrocytes into A2 astrocytes in ischemic rats [49].

Finally, Li et al. (2020) developed a model of chronic post-surgical pain causing the activation of microglia at an early stage and, 14 days later, activating astrocytes, mainly A1 astrocytes [43]. The authors observed that intrathecal injection of minocycline (a non-specific microglial inhibitor) alleviated mechanical allodynia and reverted the A1/A2 astrocyte ratio. It also increased the expression of chemokine receptor 7 (CXCR7) and the activation of the PI3K/AKT pathway (Figure 1). Similar results were obtained after intrathecal injection with AMD3100 (a CXCR7 agonist). However, administering LY294002 (a specific PI3K inhibitor) inhibited the transformation of A1 astrocytes into A2 astrocytes induced by minocycline and AMD3100. Therefore, Li et al. concluded that microglia negatively regulate the CXCR7/PI3K/AKT pathway and cause A1 astrocyte reactivity. They also determined that positive regulation of this pathway promotes A2 astrocytes [43].

These findings have important implications for understanding astroglial responses in neurodegenerative conditions and developing therapeutic strategies to modulate astrocyte reactivity and promote neuroprotective responses. However, further research is needed to fully elucidate the mechanisms that regulate astroglial responses and how these may influence the progression of neurodegenerative diseases. A complete understanding of signaling pathways will provide tools for developing efficient astrocyte-targeting therapies to improve the quality of life of patients suffering from neurodegenerative diseases.

## 3. Structural and Molecular Markers of Reactive Astrocytes

Astrocytes have historically been considered a homogeneous cell population despite their structural and functional complexity [50]. However, recent studies have shown that several structural changes, physiological properties, and responses to harmful insults depend on the brain region [11]. Since the beginning of the last century, at least two populations of astrocytes have been classified as protoplasmic and fibrous based on their shape [50].

For a long time, research on astrocytes has relied upon immunostaining techniques that target unspecific structural markers, such as the GFAP, vimentin, or the S100 calcium-binding protein β (S100β). However, these techniques have certain limitations: (1) whole cell morphology is not revealed, and it cannot fully distinguish between individual cells [51,52]; (2) GFAP, vimentin, and S100β are universal markers for astrocyte reactivity [7], which would not allow identifying the structural differences between populations. 

The shape of astrocytes depends on their localization in the brain parenchyma and their response to a pathological condition. Reactive protoplasmic astrocytes in the gray matter possess hypertrophic ramifications that increase in quantity and length in a pathological state [32]. In contrast, fibrous astrocytes in the white matter show bimodal reactivity, initially acquiring an amoeboid shape by retracting their prolongations that later return to the shape of the non-reactive state [53]. Furthermore, structural changes in astrocytic have been observed during the neurodegeneration process, such as in Alzheimer’s disease (AD). For example, hypotrophic astrocytes are observed in the early stages of AD, whereas hypertrophic astrocytes appear later [51]. The mechanisms involved in these structural differences are not fully elucidated [54,55]. It has been suggested that cell-adhesion molecules of the neuroligin family, such as NL1-3, influence astrocyte morphogenesis through interactions with other neural cell-adhesion molecules known as neurexins [56]. It would mean that astroglial morphogenesis depends on direct contact with neuronal processes or the modulation of transcription factors, like NF-κB and Notch pathway, by inflammatory and anti-inflammatory stimuli [57].

Differences in astrocyte transcriptome in response to injury have been useful in identifying cellular markers that allowed classifying astrocytes as A1 and A2 based on Liddelow’s work [8]. However, most markers of reactive astrogliosis, such as GFAP, are not specific to a particular phenotype because they are overexpressed in all reactive astrocytes. Ideally, two specific markers should be quantitatively analyzed together [58]. An example is the combination of complement C3 [14] and guanine nucleotide-binding protein 2 (GBP2) [59], which have been widely used as specific reactivity markers for A1 astrocytes, as they are overexpressed in nervous system lesions that involve the release of pro-inflammatory cytokines and are not expressed in injuries associated with the release of neurotrophic factors. However, exceptions to this combination have been reported. In human samples of sporadic Creutzfeldt–Jakob disease, a prion disease previously known as transmissible spongiform encephalopathy, reactivity to GBP2 but not to C3 has been found, thus indicating that GBP2 is a better marker for A1 astrocytes at least for this disease [59].

On the other hand, the calcium-binding protein A10 (S100A10) [14] and pentraxin 3 [59] have been widely used as A2-specific reactivity markers. In addition, it should be considered that there is also variability in the molecular expression of the same astrocytic phenotype in different neurological diseases [58]. The variation in astrocyte markers reinforces the idea that astrocytes should be described based on their structural, transcriptomics, proteomics, morphology, and functional features [12].

Some articles have reported structural differences between phenotypes of reactive astrocytes. Indeed, it has been described in the rat hippocampus that astrocytes of the A1 phenotype (GFAP+ and C3+ astrocytes) display longer dendrites after infrasound exposure [60]. In contrast, astrocytes of the A2 phenotype (GFAP+ and S100A10+ astrocytes) show hypertrophic cell bodies with fewer dendrites [60]. Another study in a rat model of ischemic heart failure found that A1 phenotype astrocytes (increased *C3* and *Serping1* and decreased transcription of *Tm4sf1* and *Sphk1*) in the central amygdala and hypothalamus paraventricular nucleus exhibit a decrease in surface area, cell volume, filament length, and process complexity and increased soma volume in a pro-inflammatory context (increased *TNFα*, *IL-1β*, and *IL-6* transcription) compared to non-reactive astrocytes (double positive for GFAP and glutamine synthetase) [61]. In contrast, another study in a traumatic brain injury rat model found that cortex-neurotoxic astrocytes (GFAP+ and C3+ astrocytes) show highly complex arborization characterized by numerous branches and branching points, as well as increased process length and complexity, compared to negative C3 astrocytes [62]. These conflicting findings highlight that it is unclear whether these structural changes translate different glial functions or how they reflect the pro-/anti-inflammatory state due to their dependence on the brain region, the noxious stimulus, and the type of disease. Therefore, further experiments are needed to accurately assess the structure of astrocytic states in different diseases, brain regions, and temporal patterns. These studies should also be conducted with functional assays to correlate the structural changes with functions of astrocyte phenotype.

The mechanisms by which astrocytes exert their neuroprotective or neurotoxic effects are not fully understood yet. Various molecules listed in Table 1 have been identified as possible mediators based on their autocrine/paracrine secretion and modulation of pro- and anti-inflammatory pathways, cytokine secretion, and cell recruitment, depending on the stimulus (Figure 1).

The astrocytes are crucial in the pathogenesis of neurodegenerative diseases, given that they participate in neuronal physiology, neurotransmission, and inflammatory responses. From the pathological point of view, Table 1 summarizes several astrocytic mediators that are highly expressed in neuroanatomic regions affected by common neurodegenerative diseases, such as the hippocampal formation in AD, the midbrain in Parkinson’s disease (PD), the basal ganglia in Huntington’s disease (HD), and the spinal cord in amyotrophic lateral sclerosis [63]. Interestingly, around 75% of the identified molecules are highly expressed in the cerebral cortex, which is frequently affected in the early or late stages of various neurodegenerative diseases [64,65], as well as in different neurological disorders elicited by psychiatric, ischemic, metabolic, infectious, and traumatic insults [66,67,68,69,70]. Promising therapeutic approaches targeting intracellular signaling pathways to increase the neuroprotective response or decrease the neurotoxic effect of reactive astrocytes are also analyzed.

**Table 1:** Neurotoxic and neurotrophic mediators associated with astrocytes.

Mediator	Brain Expression *	Physiological Roles **	Neuropathological Roles	Model/Astrocyte Description ***	Refs.
Neurotoxic mediators					
CD49f	TH, 25; HY, 20.9; MO, 20.8; SC, 20.5; CTX, 20.3.	Essential for NRG1-ERBB signaling (glutamatergic circuit).	Failure in phagocytosis, glutamate uptake, neuronal maturation, myelination, and neurotransmission.	TNFα, IL-1α, and C1q induced iPSC-derived astrocytes (A1-like reactive state; C3+).	[71,72]
SERPINA3	HY, 320; CTX, 293.8; MO, 196.3; MB, 183.1; TH, 176.3.	Inhibits the activity of proteases (cathepsin and chymases).	Regulates the expression of NF-κβ. Promotes macrophage migration and BBB dysfunction, as well as the formation of protein plaques.	TNF-induced iPSC-derived BBB co-culture model (inflammatory reactive state).	[73,74,75]
C1q	CTX, 135.3; WM, 131.6; MO, 126.7; P, 110.3; MB, 110.1.	Involved in inflammation and infection, ribosome biogenesis, protein synthesis, regulation of apoptosis, and transcription.	Implicated in excitatory and inhibitory synapse elimination. Causes activation of pro-inflammatory microglia. Prevents differentiation and maturation of oligodendrocytes.	Reactive astrocytes (GFAP+) in the *P301S Tau* transgenic mouse model.	[76,77]
C3	MO, 143.3; WM, 121.9; P, 116.4; TH, 103.4; MB, 99.2.	Participates in the activation of the complement system. Chemoattractant for neutrophils.	Modulates microglial phagocytosis. Dysregulation of intraneuronal Ca^2+^ homeostasis and excitotoxicity. Disrupts dendritic morphology.	TNFα-induced knock-out *IκBα* transgenic mice astroglia (GFAP+).	[78,79]
GM-CSF	CTX, 0.1; HP: 0.1; AMY, 0.1; MO, 0.1; WM, 0.1.	Stimulates the growth and differentiation of hematopoietic precursor cells.	Induces proliferation of microglia and neuronal network dysfunction. Promotes the migration of inflammatory cells across the BBB.	Non-obese diabetic mice (model for secondary progressive multiple sclerosis) pro-inflammatory active astrocytes (GFAP+).	[80,81,82]
CXCL10	P, 10.8; MO, 7.4; SC, 3.4; TH, 2.6; CTX, 2.6.	Chemotaxis, differentiation, and activation of peripheral immune cells, regulation of cell growth, and apoptosis.	Encourages immune cell infiltration and contributes to establishing a pro-inflammatory CNS environment.	Non-obese diabetic mice (model for secondary progressive multiple sclerosis) pro-inflammatory active astrocytes (GFAP+). Pro-inflammatory reactive astrocytes.	[80,83,84]
CCL2	TH, 43.4; MO, 32.7; CTX, 26.8; WM, 25.3; P, 21.9.	Chemotactic response and mobilization of intracellular calcium ions.	Controls the recruitment of perivascular leukocytes into the CNS and shifts to an inflammatory phenotype. Potentiates the activation of astrocytes and microglia, demyelination, and axonal loss.	EAE mouse spinal cord reactive astrocytes (GFAP+). TNFα-stimulated primary mouse astrocytes (GFAP+).	[80,85,86,87]
IL-17	WM, 79.8; BG, 79.7; SC, 79.6; MO, 79.1; AMY, 71.9.	Involved in antimicrobial host defense and maintenance of tissue integrity.	Recruits NF-κβ activator 1 and subsequent pro-inflammatory cytokine production. Induces demyelination.	*Angiostrongylus cantonensis* mouse active A1 astrocytes (GFAP+, S100β+, C3+)	[80,88,89]
TrkB	HYP, 230; TH, 222.1; MB, 218.9; AMY, 201.9; CTX, 190.6.	Regulation of neuronal survival, proliferation, migration, differentiation, and plasticity.	Promotes excessive NO production and neuronal dysfunction or death by inducing excitotoxicity.	EAE mouse spinal cord astrocytes (GFAP+) and human multiples sclerosis lesion astrocytes (GFAP+). Hippocampal astrocytes (GFAP+) in the lithium-pilocarpine temporal lobe epilepsy mouse model.	[90,91]
NLRP3	WM, 3.4; P, 2.1; MO: 2; SC, 1.9; TH, 1.6.	Mediates NLRP3 inflammasome activation.	Involved in neuroinflammation and mitochondrial dysfunction.	Hippocampal A1 astrocytes in the chronic intermittent hypoxia rat model (GFAP+, C3+, increased synaptic branches, junctions, end-point voxels, and decreased branch length).	[92,93,94]
ELOVL1	WM, 165.7; MO, 152; P, 140.7; CTX, 116.9; BG, 115.6.	Participates in the LCFA elongation cycle.	Saturated LCFA mediates astrocyte-induced toxicity through lipoapoptosis (PERK pathway).	Neurotoxic reactive astrocytes in the TNF-α, IL-1α, and C1q-induced primary murine model.	[95]
FABP7	CTX, 366.1; CB, 228.1; BG, 210.5; HP, 198.7; MO, 188.	Involved in fatty acid metabolism and establishing the radial glial fiber system in the developing brain.	Promotes the NF-κB-driven pro-inflammatory response, which is detrimental to motor neuron survival.	Spinal cord pro-inflammatory/neurotoxic astrocytes (GFAP+, FABP7+) in the hSOD1-linked ALS mouse model.	[96]
Notch 1	BG, 16.1; WM, 14.0; TH, 13.9; MO, 13.5; MB, 12.3.	Regulates differentiation, proliferation, apoptosis, neurogenesis, gliogenesis, and neuritogenesis.	Promotes the secretion of pro-inflammatory neurotoxic factors, neuronal apoptosis, and axonal damage.	Contusive spinal cord injury rat neurotoxic A1 astrocytes (GFAP+, C3+, NICD+) and induced (TNFα, IL-1α, and C1q) primary astrocytes (A1 phenotype).	[97]
SRR	HYP, 13.4; CTX, 13.1; P, 12.7; TH, 12.5; CB, 12.5.	Catalyzes the synthesis of D-serine, a key coagonist with glutamate at NMDA receptors.	May contribute to their neurotoxic effects by activating extra-synaptic NMDA receptors.	Primary mouse reactive astrocytes (GFAP+, C3+).	[98]
HMGB1	CTX, 362.4; WM, 308.2; P, 286.2; HYP, 282.9; BG, 282.2.	It is a DNA chaperone involved in replication, transcription, chromatin remodeling, DNA repair, and genome stability.	Promotes the expression of pro-inflammatory cytokines through RAGE signaling.	Hippocampal astrocytes (GFAP+) and primary microglia-stressed A1 astrocytes in mice with sepsis-associated encephalopathy.	[99]
Kir 6.2	CB, 12.4; CTX, 11.9; BG, 8.3; P, 8.2; MO, 8.	Subunit of ATP-sensitive potassium channels.	Mediates mitochondrial fragmentation, resulting in its malfunctioning.	Reactive A1 neurotoxic astrocytes (GFAP+, C3+) in an LPS-induced PD mouse model.	[100]
Lcn2	CTX, 0.5; P, 0.3; MO, 0.3; BG, 0.1; TH, 0.1.	Iron-trafficking protein involved in apoptosis and innate immunity	Contributes to neuronal loss, pro-inflammatory cytokine expression, and immune cell infiltration	Pro-inflammatory astrocytes in LPS-induced primary spinal cord mice; TNFα, IL-1α, and C1q primary spinal cord A1 phenotype astrocytes. Contusive spinal cord injury rat astrocytes (GFAP+)	[101,102]
Neurotrophic mediators					
FZD1	MB, 6.2; CB, 5.8; HF, 4.7; TH, 3.4; HY, 2.9.	Involved in the Wnt/β-catenin signaling pathway, which is associated with neuroprotection.	An alteration of Wnt, a pathway controlled by FDZ1, contributes to Tau hyperphosphorylation, memory impairment, and increased Aβ production through GSK3 hyperactivity. Mediates midbrain dopaminergic neurodevelopment as well as its recovery after insults.	MPTP-induced PD C57BL/6 mice activated astrocytes of the ventral midbrain (GFAP+) and primary astrocytes from mouse ventral midbrain and aNPCs from subventricular zone and midbrain co-culture (treated with MPTP and the Wnt inhibitor Dkk-1).	[103,104]
ARG1	CB, 1.0; CTX, 0.5; MO: 0.4; P, 0.3; HP, 0.3.	Implicated in neuronal growth/regeneration and adaptive/innate immune responses.	The astrocytic urea cycle exerts opposing roles of beneficial Aβ detoxification and detrimental memory impairment in AD.	Aβ primary astrocyte cultures and reactive astrocytes from postmortem hippocampal samples of AD patients (GFAP+, ARG1+) and transcriptome data (KEGG pathway analysis).	[105]
Nrf2	WM, 91.8; CTX, 86.7; P, 84.5; BG, 83.9; MO, 82.	Immune system maintenance. Upregulates genes that promote glutathione synthesis. Reduces the expression of pro-inflammatory cytokines.	Coordinates the upregulation of antioxidant defenses. Its deficiency promotes oxidative stress and abnormal neuroinflammation by the upregulation of pro-inflammatory cytokines and, therefore, neurodegeneration.	Familial ALS mouse models overexpressing Nrf2 astrocytes that induce neuroprotection (GFAP+, hPAP+) and primary astrocyte–motor neuron co-cultures.	[106,107,108]
SPHK1	TH, 8.5; P, 4.5; MB, 4.1; MO, 4.0; AMY, 4.	Regulates neuroinflammation response. Stimulates the activation of NF-kβ for IL-17 synthesis. Contributes to cellular survival.	Reduction of SphK1 expression can lead to defective microglial phagocytosis and dysfunction of inflammation resolution due to decreased secretion of specialized pro-resolving mediators. Sphk1 binds its receptor and facilitates GDNF-induced enhancement in the transcription of GAP43, a key protein in axons.	6-OHDA hemiparkinsonian mouse (injected with desipramine for dopaminergic protection) protective A2 astrocytes (GFAP+, neuroprotective genes+).	[109,110]
MFGE8	CTX, 61.8; CB, 56.4; MO, 55.9; P, 55.6; WM, 55.6.	Mediates angiogenesis and the anti-inflammatory response through phagocytosis and pro-inflammatory cytokine downregulation.	Promotes neural stem cell proliferation and migration toward ischemic brain tissues. Increases microglial phagocytosis of myelin debris and promotes remyelination.	MFGE8 overexpressing KO/BCAS mouse (AAV vector) astrocytes (GFAP+; CD45-/GLAST1+) and primary astrocyte-OPC neuron cultures	[111,112]
BDNF	HP, 8.2; CTX, 5.3; CB, 3.3; M, 3.3; P, 2.5.	Contributes to survival and differentiation of neuronal development, synaptic plasticity, and memory formation.	A decrease or polymorphism of BDNF is associated with cognitive decline, tau phosphorylation, synapse loss, and neurodegeneration. Promotes dendrite outgrowth and spine density	5xFAD mice that overexpress BDNF reactive astrocytes (GFAP +).	[113,114]
TSP1	TH, 14.3; CTX, 11.3; P, 10.8; MO, 10.8; MB, 4.4.	Involved in angiogenesis and promotion of cell adhesion. Contributes to neuroprotection against Aβ.	Regulates signaling pathways involved in inflammation. Determines peripheral Aβ homeostasis.	*P301S Tau* or C57 mouse-derived neuron–astrocyte co-cultures and neuron culture treated with ACM and anti-TSP1 antibody or recombinant mouse TSP1.	[115]
TGF-β1	MO, 54.2, TH, 43.4, SC, 42, WM, 40, P, 39.2	Plays an essential role in neuronal survival and modulates the expression and activation of other growth factors, like interferon-gamma and TNFα.	Any alteration in TGF-β1 signaling contributes to AD through reduced phosphorylation of Smad2/3 and downregulation of TGF-β1 type II receptor expression.	Reactive astrocytes (GFAP+) in SBE-LucRT mice to measure TGFβ signaling after stroke by occlusion of a distal middle cerebral artery.	[116,117]

Abbreviations: 6-OHDA: 6-hydroxydopamine; Aβ: amyloid beta; AAV: adeno-associated virus; ACM: astrocyte-conditioned medium; AD: Alzheimer’s disease; ALS: amyotrophic lateral sclerosis; aNPCs: adult neural stem/precursor cells; ARG1: Arginase 1; ATP: adenosine triphosphate; BBB: blood–brain barrier; BCAS: bilateral carotid artery stenosis; BDNF: brain-derived neurotrophic factor; C1q: complement component 1q; C3: complement C3; CCL2: C-C motif chemokine ligand 2; CD49f: integrin subunit alpha 6; CNS: central nervous system; CXCL10: C-X-C motif chemokine ligand 10; Dkk-1: Dikkhopf-1; EAE: experimental autoimmune encephalomyelitis; ELOVL1: elongation of very-long-chain fatty acid protein 1; ERBB: EGF receptor family; FABP7: brain-type fatty acid-binding protein 7; FZD1: frizzled class receptor 1; GAP43: growth associated protein 43; GLAST1: glutamate aspartate transporter 1; GM-CSF: granulocyte–macrophage colony-stimulating factor 2; GSK3: glycogen synthase kinase 3; HMGB1: high-mobility group protein B1; hPAP: human placental alkaline phosphatase; hSOD1: human superoxide dismutase 1; IκBα: nuclear factor of kappa light polypeptide gene enhancer in B-cell inhibitor alpha; IL-1α: interleukin 1 alpha; IL-17: interleukin 17; iPSC: human-induced pluripotent stem cell; KCNJ11 (kir6.2): ATP-sensitive inward rectifier potassium channel 11; KO: knock out; LCFA: long-chain fatty acid; Lcn2: Lipocalin 2; LPS: lipopolysaccharide; MFG E8: milk fat globule-epidermal growth factor 8; MPTP: 1-methyl-4-phenyl-1,2,3,6-tetrahydropyridine; NF-κβ: nuclear factor kappa-light-chain-enhancer of activated B cells; NICD: notch intracellular domain; NLRP3: nucleotide oligomerization domain (NOD)-like receptor protein 3; NMDA: N-methyl-D-aspartate; NO: nitric oxide; Notch 1: neurogenic locus notch homolog protein 1; Nrf2: nuclear factor erythroid 2-related factor 2; NRG1: Neuregulin-1; OPC: oligodendrocyte progenitor cell; PD: Parkinson’s disease; PERK: protein kinase R-like endoplasmic reticulum kinase; RAGE: receptor for advanced glycation end products; S100β: S100 calcium-binding protein B; SBE-lucRT: Smad-responsive luciferase promoter; SERPINA3: Alpha 1-antichymotrypsin; Smad: mothers against pentaplegic homolog; SPHK1: sphingosine kinase 1; SRR: serine racemase; TGF-β: transforming growth factor beta; TNFα: tumor necrosis factor alpha; TrkB: tyrosine receptor kinase B; TSP1: Thrombospondin 1; Wnt: wingless-related integration site. Brain regions: AMY: amygdala; BG: basal ganglia; CB: cerebellum; CTX: cerebral cortex; HP: hippocampus; HY: hypothalamus; MO: medulla oblongata; MB: midbrain; P: Pons; SC: spinal cord; TH: thalamus; WM: white matter. * Gene expression by brain region was obtained from the Human Protein Atlas (http://www.proteinatlas.org/, accessed on 13 May 2024). ** Data source: UniProt.org (accessed on 13 May 2024). *** Terminology used to describe astrocytes in the cited articles.

## 4. Roles of Reactive Astrocytes in Neurodegenerative Diseases and Therapeutic Approaches

This section discusses how astrocyte reactivity is involved in the progression of common neurodegenerative diseases. Furthermore, we identify potential therapeutic strategies associated with neurotoxic and neuroprotective mediators in different signaling pathways of astrocytes (Figure 1 and Table 2).

### 4.1. Alzheimer’s Disease

AD is the most common neurodegenerative disorder, affecting approximately 50 million patients worldwide [118]. Histopathologically, AD is characterized by amyloid plaques formed by the extracellular deposition of amyloid-beta peptide (Aβ) and neurofibrillary tangles produced by hyperphosphorylation of the microtubule-associated Tau protein [119]. Aβ oligomers activate microglia, releasing pro-inflammatory cytokines that ultimately lead to Tau protein hyperphosphorylation and aggregation. Tau release upon neuronal death also causes microglial activation, thus generating a vicious cycle that ends in neurodegeneration [119,120]. Astrocytes are largely involved in amyloid pathology since they are abundant in the CNS, but their role in AD has been less associated with neurodegeneration than that of microglia [121]. However, along with the connection between astrocytes and AD, their roles in AD development and progression are currently gaining more attention. 

Studies on AD brains have shown that reactive astrocytes accumulate around Aβ deposits [122]. It has also been demonstrated that after exposure to Aβ, astrocytes polarize to the A1 state and release a series of cytokines, such as IL-1β or IL-6, NO, ROS, and glutamate [8,123,124]. Growing evidence suggests that reactive astrocytes are primarily located in the microenvironment of amyloid plaques [125,126,127]. Interestingly, Olabarria et al. (2010) observed astroglial hypertrophy around the plaques and astroglial atrophy far from the plaques as a generalized process [126]. Moreover, Habib et al. have identified a unique reactive specific astrocyte subtype of AD, named disease-associated astrocyte (DAA). DAAs appear in the early stages of the disease, mainly localized around Aβ plaques in both the hippocampus and subiculum. These regions are adversely affected by AD [128]. To date, the involvement of A2 astrocytes in AD has not been reported. Future research should focus on analyzing the different astrocytic phenotypes in affected areas from the early to late stages of the disease.

Proposed therapeutic approaches associated with astrocyte reactivity (Table 2) involve preventing the secretion of microglial cytokines, such as IL-1α, TNFα, or C1q, known to trigger astrocytic differentiation to the neurotoxic phenotype [129]. Some drugs that have been shown to influence this mechanism are agonists of the glucagon-like peptide-1 (GLP-1) receptor (GLP-1R) (GLP-1RA), TNFα inhibitors (i-TNFα), C1q inhibitors, and IL-1α inhibitors, among others. 

GLP-1RAs, an approved treatment option for type 2 diabetes mellitus, have autonomic and neuroendocrine regulatory actions associated with neuroprotective effects [128]. GLP-1R is expressed in various areas of the CNS that are closely related to memory and learning [130]. Several studies have indicated that GLP-1RA improves cognitive dysfunction in vivo and protects astrocytes in vitro, potentially by inhibiting the NLRP3 inflammasome pathway [131,132]. Another hypothesis is that GLP-1RAs influence brain metabolism and its action on astrocytic aerobic glycolysis, during which GLP-1 increases astrocytic glycolytic capacity via phosphatidylinositol-3-kinase (PI3K/Akt). NLY01, a long-lasting GLP-1RA, is proposed to block neurotoxic astrocytes. It effectively penetrates the BBB and inhibits reactive microglia-induced astrocytes while preserving neuronal viability [130,133,134].

Dimethyl itaconate (DI), an itaconate derivative, reduces neurotoxic reactive astrocytes and inhibits inflammasome assembly, reducing caspase-1 cleavage and IL-1β levels. Furthermore, DI attenuates NF-κB phosphorylation and exerts a neuroprotective effect by reprogramming astrocytes from an A1 neurotoxic state to an A2 neuroprotective state [135]. Microglial activation of TLR4, which triggers NF-κB signaling, causing a specific A1 astrocyte response in brain tissue, was observed in *APP/PS1* transgenic (Tg) mice, which is a model of AD. The impact of treatment with Fasudil, an inhibitor targeting the RhoA/ROCK signaling pathway associated with inflammation and oxidative stress, has also been investigated. Fasudil suppressed microglial activation and promoted the expression of the A2 astrocyte phenotype through *TLR4* and *NF-κB* downregulation [136].

Moreover, there are other drugs with potential effects on the neurotoxic effects of reactive astrocytes in AD, such as etanercept (i-TNFα), anakinra (recombinant IL-1α antagonist), non-steroidal anti-inflammatory drugs (NSAIDs), and C3 or C1q inhibitors [137], among others. Cornuside, for example, is an iridoid glycoside from *Cornus officinalis* that modulates memory deficits in Tg mice used as AD models. This glycoside could suppress the activation of the NF-κB pathway, upregulated in the cortex and hippocampus of AD mouse models, through *IκBα* downregulation and p65 protein upregulation. Cornuside was proposed as a regulator of astrocyte reactivity that enhanced synaptic plasticity and alleviated cognitive decline through the NF-κB signaling pathway in an AD mouse model [138].

On the other hand, applying specific cytokines has been considered a potentially helpful approach for astrocyte reactivity to promote the synthesis of neuroprotective mediators (Table 2). These cytokines include tissue inhibitors of metalloproteinase-1 (TIMP-1), intercellular adhesion molecule-1 (ICAM-1), TGF-β, IL-1, and interferon-β (IFN-β), among others. Saha et al. (2020) demonstrated that the intracerebroventricular administration of recombinant exogenous TIMP-1 in rats infused with Aβ reduced this peptide and apoptosis in the hippocampus and other cortical regions. In addition, this drug decreased cognitive dysfunction and promoted the activation of neuroprotective astrocytes through Akt pathway activation [139].

While astrocyte-targeted therapeutic options appear promising, more research is needed to assess their efficacy and safety. Some drugs mentioned in this section can potentially regulate astrocyte reactivity, but more studies are required to fully understand the effects of astrocytes. Understanding the complex interplay between astrocytes, microglia, and other factors involved in neurodegeneration will be critical to advancing effective treatments for AD. While these drugs could alleviate AD symptoms, approaches to stop neurodegeneration and promote the correct structural and functional recovery of neural circuitry damaged by AD are needed for an efficient treatment against AD. 

### 4.2. Parkinson’s Disease

PD is the second most common neurodegenerative disorder, with an estimated worldwide prevalence of 6.2 million patients [140]. Histopathologically, it is characterized by pathological α-synuclein (α-syn) aggregates, neuroinflammation, and neurodegeneration of the dopaminergic nigrostriatal system [141,142]. It has been suggested that α-syn plays a significant role in astrocytic activation, attributed to the presence of α-syn aggregates within reactive astrocytes [143]. It should be noted that while intracellular α-syn has been detected in reactive astrocytes, the clearance of extracellular α-syn is reduced, implying that astrocytes may face challenges in effectively breaking down ingested α-syn [143]. Also, it has been suggested that A1 reactive astrocytes are induced by α-syn–activated microglia [129].

Oxidative stress, impaired lysosomal degradation mechanisms, and mitochondrial dysfunction have been associated with pathological α-syn accumulation [144,145]. Research suggests that this abnormal accumulation would result in a change from A2 astrocytes to A1 reactive astrocytes, indicating that increasing the A1/A2 ratio of reactive astrocytes drives the abnormal aggregation and spread of α-syn and, therefore, PD progression.

On the other hand, extracellular α-syn could activate microglia through TLR2 and TLR4 [146], acting as damage-associated molecular patterns and inducing the activation of NF-κB [92,147] and the production of pro-inflammatory cytokines [92]. In this way, the role of α-syn in astrocytes is highlighted since its continuous misfolding and resistance to degradation could generate a perpetual state of reactivity. 

Luna-Herrera et al. (2020) reported that the A1 reactive astrocyte response was associated with the loss of dopaminergic neurons in the *substantia nigra pars compacta* (SNpc) in a PD animal model [148]. In postmortem studies, Liddelow et al. demonstrated the presence of A1 reactive astrocytes in the brains of PD patients [8]. Likewise, Valenzuela et al. showed that intranigral administration of LPS induced the A1 neurotoxic phenotype in a progressive and sustained manner associated with the progression of dopaminergic neurodegeneration. Moreover, in this PD animal model, the A2 astrocyte phenotype presents a defensive response to injury in the brain [92].

Concerning therapeutic approaches, identifying reactive astrocytes within the SN in both patients and preclinical models of PD is a crucial element (Table 2). The use of NLY01 in PD models has a neuroprotective effect through the direct prevention of astrocyte reactivity mediated by the blockade of microglia and inhibition of the release of IL-1α, TNFα, and C1q [129]. However, no further studies have contributed to improving the evidence of the blocking effect of NLY01 on reactive astrocytes. Interestingly, NLY01 is currently being clinically evaluated [NCT04154072] to assess the safety, tolerability, and efficacy of NLY01 in subjects with early untreated PD.

Statins, which are primarily used to inhibit cholesterol synthesis, are another class of drugs with the potential to influence astrocyte reactivity by inhibiting the enzyme 3-hydroxy-3-methyl-glutaryl-coenzyme A reductase (HMG-CoA reductase). Statins have shown beneficial effects for managing neuroinflammatory conditions [149,150]. It has been identified that simvastatin, a lipophilic statin capable of crossing the BBB, can modulate the activity of the nuclear receptor subfamily A group 4 member 2 (NR4A2, also known as NURR1) in reactive astrocytes. NURR1 belongs to a group of orphan nuclear receptors with broad physiological actions. For example, it antagonizes the signaling of the NF-κB pathway of macrophage and microglia pro-inflammatory genes, thereby influencing astrocyte reactivity [151,152]. Through these mechanisms in reactive astrocytes, it is proposed that simvastatin decreases the production of IL-6 and TNFα and their amount in the SNpc, which prevents the loss of dopaminergic neurons with consequent improvement in behavioral tests of preclinical models [151,153]. Simvastatin was tested in a clinical trial to evaluate its neuroprotective effect in patients with moderate severity of the condition. The trial was a double-blind, randomized, placebo-controlled futility study, and the primary outcome was a change in the Unified Parkinson’s Disease Rating Scale (MDS-UPDRS) Part III motor subscale score in the OFF-medication state (OFF state). However, study results have not been submitted to ClinicalTrials.gov [154]. Future research is required to better understand and develop astrocyte-targeted therapeutic strategies in PD, highlighting the role of α-syn in astrocyte activation.

### 4.3. Huntington’s Disease

HD is the most common of the dominant hereditary neurodegenerative diseases. It is characterized by neuropsychiatric and motor symptoms such as chorea, akinesia, bradykinesia, hypokinesia, and progressive cognitive impairment [155]. HD typically begins in adulthood and follows an irreversible progression of 15 to 20 years, ultimately leading to death [156]. It is caused by an autosomal mutation of the *Huntingtin* gene (*HTT*), with full penetrance, when the CAG codon exceeds 40 repeats [157]. The amyloidogenic mutant huntingtin (mHtt) forms soluble fragments and aggregates that are toxic to neurons, particularly the medium-spiny neurons in the striatum [13]. The presence of mHtt and neurodegenerative processes in the striatum can trigger immunological responses of microglia and astrocytes [158]. In this context, it was demonstrated in various Tg mouse models that mHtt inclusions trigger microglial activation [159], which promotes the expression in astrocytes of pro-inflammatory genes through NF-κB activation [158]. Furthermore, the increased reactivity of A1 astrocytes is linked to a neurotoxic phenotype in the brains of HD patients because elevated counts of C3 immunoreactive astrocytes have been found in the caudate nucleus [8,160].

Diaz-Castro et al. (2019) suggest that A1 and A2 astrocyte activity may not be observed in all areas of the HD brain but only in areas affected by HD [157]. Using RNAseq, these authors evaluated astrocyte reactivity genes in both R6/2 and Q175 mouse models of HD. No astrocyte reactivity occurred in the early stages of HD, only in the late stages where they found increased gene activity related to A1 astrocytes [157]. However, these researchers suggest that astrocyte reactivity is unlikely to contribute to neurodegeneration in the early stages. 

Likewise, in a rat model of HD induced by 3-nitro propionic acid, researchers observed motor dysfunctions, neurological disorders, and damage in the striatum [161]. The study reported a significant increase in A1 astrocytic levels in the striatum, hippocampus, and cerebellum through histological and molecular analysis for GFAP, C3, TNFα, and IL-1α [161]. Subsequently, the same working group reported the therapeutic use of kaempferol in this HD model (Table 2) since the treatment decreased the reactivity of A1 astrocytes [162]. As an attractive astrocyte-focused therapeutic strategy, kaempferol has recently gained relevance for its anti-inflammatory and anti-neuroinflammatory actions [162]. The effect is mainly attributed to its ability to inhibit phospholipase A2, lipoxygenase, cyclooxygenase, and NO synthesis by inhibiting the inducible NO synthase (iNOS) enzyme [162]. These actions are attractive pharmacological targets in astrocytes since kaempferol can cross the BBB [163]. Although the exact mechanism of its antioxidant and anti-inflammatory actions is not fully known, it is believed that the inhibition of the NF-κB pathway and decrease in pro-inflammatory cytokines IL-1α, TNFα, and C1q are responsible for kaempferol’s neuroprotective effects. These effects may inhibit astrocyte reactivity differentiation in preclinical models of HD [162]. Understanding the complex interplay between glial cells and their activation patterns holds promise for future therapeutic strategies targeting astrocytes in HD.

### 4.4. Multiple Sclerosis

Multiple sclerosis (MS) is an inflammatory and neurodegenerative disease and the leading cause of non-traumatic disability worldwide [164,165]. According to the MS Atlas by the International Federation of MS, this disease affected around 2.8 million people in 2020 [166]. Neurologically, MS patients experience episodes of motor dysfunction as well as sensory symptoms and optic neuritis. 

MS is characterized by neuronal demyelination, axonal degeneration, and reactive gliosis in white and gray matter in the brain and spinal cord and by the infiltration of macrophages and T and B lymphocytes [167,168]. The interaction between infiltrating T cells and astrocytes is pivotal [169]. This interaction occurs directly with T cells binding to astrocytes via major histocompatibility complex molecules, leading to immune response activation and inflammatory factor release [170,171]. Additionally, indirect mechanisms involve T cells releasing cytokines like IL-17 and IL-6, thus prompting astrocyte reactivity [170,172]. Ultimately, these interactions create an inflammatory environment that contributes to myelin and neuronal damage in MS.

In MS, astrocytes are transformed to the A1 phenotype [8], which could secrete several neurotoxic cytokines such as TNFα, IL-1β, and IL-6, leading to the formation of a pro-inflammatory environment in the CNS, myelin damage, and deterioration of the remyelination process [173].

Several reports indicate that specific microRNAs play a vital role in the processes of demyelination and remyelination in MS [174,175]. Upregulation of miR-155 inhibited myelin repair by promoting a pro-inflammatory microenvironment in the CNS [176]. Furthermore, it was confirmed that miR-155 promotes pro-inflammatory cytokine production in astrocytes [176]. Interestingly, it has been suggested that the A1 phenotype induces oligodendrocyte death and inhibits differentiation and maturation of oligodendrocyte precursors, thus exacerbating demyelination and preventing myelin repair [8]. In MS, oligodendrocytes are the target of inflammatory and immune attacks, and their gradual death leads to demyelinated lesions and remyelination failure [177].

On the other hand, A2 astrocytes enhance the maturity of oligodendrocyte precursor cells and protect against the progression of white matter lesions [178]. MiR-155 may regulate the transition between A1 and A2 reactive astrocytes, making it a potential treatment target for myelin repair in MS [179].

The presence of reactive astrocytes has been evidenced and associated with demyelinating lesions and neurodegeneration zones [180]. These findings suggest a link between NLRP3 inflammasome activation in microglia and the conversion of astrocytes into the neurotoxic A1 phenotype [180]. Additionally, A1 astrocyte reactivity was recently evaluated through histological assays, where an increased C3 marker perpetuates neuronal damage [179]. Similarly, an alteration in the phenotype with a hypertrophic cell body and thick processes has been recorded [181]. Current research shows that several drugs used to treat demyelinating diseases, such as MS, provide therapeutic benefits by modulating astrocytic activity, either by reducing the harmful actions of reactive astrocytes or by potentiating their beneficial effects [182,183].

Dimethyl fumarate (DMF) is a drug belonging to the group of fumaric acid esters (Table 2). It has immunomodulatory and neuroprotective properties and treats MS as a disease-modifying agent for relapsing-remitting MS [184]. Both DMF and isosorbide DMF (IDMF), a new molecule synthesized through the esterification of isosorbide with DMF, decreased C3+ reactive astrocytes by activating the nuclear factor-related erythroid factor-2 (Nrf2) pathway, the master transcription regulator of oxidative stress-related genes, including those of the NF-κB pathway and hypoxia-inducible factor 1 alpha [184,185]. Another study carried out in fetal human astrocytes identified that IDMF alters the expression of genes associated with MS, including antioxidant gene *heme oxygenase 1* and genes linked to the integrity of the extracellular matrix (*metallopeptidase inhibitor 3* and *serum matrix metalloproteinase-9*). Moreover, the same study found that IDMF downregulates mitogenic genes associated with the reactive astrocytes, such as the *intercellular adhesion molecule 1* gene. These findings suggest that the IDMF-mediated neuroprotection could be due to inhibiting astrocyte reactivity [186].

Siponimod and fingolimod (Table 2) are MS-modifying drugs approved for the treatment of secondary progressive and relapsing MS, respectively. They function as sphingosine-1-phosphate (S1P) receptor 1 antagonists and S1P receptor 5 agonists. S1P receptors are expressed in various organs and regulate functions such as lymphocyte trafficking, brain development, and vascular permeability. The S1P receptor 1 plays an essential role in neurogenesis, immune cell trafficking, and endothelial barrier function. Blocking these receptor-employing modulators in preclinical models reduces cytokine amplification and immune cell recruitment, which is the aim of MS treatment. However, S1P receptor 1 participates in glial activation and proliferation [187]. Siponimod, as an S1P receptor 1 antagonist, suppresses the activation of the NF-κB pathway and histone deacetylase enzyme. This inhibition reduces the production of pro-inflammatory cytokines IL-1β, IL-6, and TNFα at the astroglial level. Additionally, siponimod leads to S1P receptor 1 internalization in astrocytes and activates Nrf2, which is associated with antioxidant and neuroprotective responses by astrocytes [188,189,190]. On the other hand, the GLP-1RA NLY01 has shown therapeutic potential by blocking neurotoxic astrocyte conversion in preclinical models of MS [133]. 

**Table 2:** Astrocyte-targeting therapeutic approaches in distinct neurodegenerative diseases.

Model	Drug	Mechanism	Astrocytic Effect	Refs.
Alzheimer’s disease				
Transgenic mice (3-month-old 5xFAD and 7-month-old male 3xTg-AD).	NLY01	Selective blockade of Aβ-induced activation through GLP-1R activation.	Blockade of reactive astrocytes (C3+) activation through microglial blocking.	[130]
Adult male Sprague Dawley rats infused with Aβ1-42.	TIMP-1	MMP inhibitory cytokine; activation of Akt signaling pathway.	Activation of neuroprotective reactive astrocytes (TIMP+).	[139]
Transgenic mice (8-month-old 3xTg-AD).	Cornuside (7-O-Galloylsecologanol)	Activation of Akt/Nrf2 pathway and inhibition of NF-κB signaling.	Regulation of A1/A2 astrocytic phenotype from A1 to A2.	[138]
Male APP/PS1 Tg mice (8 months old).	Fasudil	Downregulation of TLR4/MyD88/NF-κB pathway.	Regulation of A1/A2 astrocytic phenotype from A1 to microglia-induced A2.	[136]
Parkinson’s disease				
PFF intrastriatal injection in 3-month-old male and female C57BL/6 mice.	NLY01	Blockade of microglia-derived factors (IL-1α, TNFα, IL-1β, IL-6).	Inhibition of reactive A1 astrocytic differentiation.	[129]
MPTP lesioned male mice ages 4–5 months old.	Simvastatin	NURR1 modulation.	Prevention of conversion into reactive A1 astrocytes in SNpc.	[191]
Male adult Sprague Dawley rats injected with LPS.			Effective inhibition of astrocytic activation.	[153]
Huntington’s disease				
Male Wistar rats, 9–10 weeks old (Murine 3-NPA model).	Kaempferol	Blockade of the NF-κB activating signaling pathway.	Inhibition of the generation of C3+ A1 reactive astrocytes.	[162]
Multiple sclerosis				
C57BL/6 J mice with EAE (9 weeks old).	NLY01	Inhibition of inflammatory microglial activity. Induction of the antioxidant Nrf2 pathway in glial cells and neurons.	Blockade of neurotoxic astrocyte conversion.	[133]
C57BL/6 mice with EAE (7 weeks old).	DMF	Activation of the antioxidant Nrf2 pathway in glial cells and neurons. Suppression of C3 deposition in astrocytes.	Blockade of C3+ reactive astrocytic conversion.	[184]
Mouse primary astrocyte-enriched cultures from newborn C57BL/6 mouse brains.	Siponimod	Suppression of the activation of the NF-κB pathway and the histone deacetylase enzyme.	Blockade of activated astrocytic responses.	[188]
In vitro human astrocytes from reprogrammed skin fibroblasts.	Siponimod	Inhibition of the NF-κB pathway and by Nrf2 factor induction.		
IFN-γ-activated astrocytes cultured from 3-day-old female Wistar rat neocortices.	Fingolimod (FTY720)	Decrease of IFN-γ- induced MHC class II expression and increase of ADR-β2 expression.	Inhibition of activated astrocytes.	[190]
Cuprizone--triggered demyelination model in female C5TBL/6J mice between 6 and 8 weeks of age.	Bu Shen Yi Sui capsule (BSYS)	Decrease in proinflammatory cytokines IL-6 and IL-1β.	Regulation of A1/A2 astrocytic phenotype from A1 (C3 and CFB+) to A2 (PTX3 and S100A10+).	[179]
		Other trials		
Primary murine astrocytes isolated from C57BL/6 pups (one day old) and stimulated with LPS.	Dimethyl itaconate	Reduce LPS-induced NLRP3 inflammasome activation and IL-1β secretion.	Reprogramming astrocytes from neurotoxic A1 (IL-1 and GFAP+) to A2 (Arg 1+) states.	[135]

Abbreviations: Aβ: amyloid beta; AD: Alzheimer’s disease; ADR-β2: β2 adrenergic receptor; Akt: protein kinase B; APC: antigen-presenting cells; APP: amyloid precursor protein; ARG1: Arginase 1; BSYS: Bu Shen Yi Sui capsule; C3: complement C3; CFB: complement factor B; DMF: dimethyl fumarate; EAE: experimental autoimmune encephalomyelitis; FTY720: fingolimod; GFAP: Glial Fibrillary Acidic Protein; GLP-1R: Glucagon-like peptide-1 receptor; IFN: interferon; IL-1α: interleukin 1α; IL-1β: interleukin 1β; IL-6: interleukin-6; LPS: lipopolysaccharide; MHC: major histocompatibility complex; MMP: matrix metalloproteinase; MPTP: 1-methyl-4-phenyl-1,2,3,6-tetrahydropyridine; MyD88: myeloid differentiation primary response protein 88; NF-κB: nuclear factor kappa-light-chain-enhancer of activated B cells; NLRP3: Nod-like receptor protein 3; 3-NPA: 3-nitropropionic acid; Nrf2: nuclear factor erythroid 2-related factor 2; NURR1: nuclear receptor related 1; PFF: α-synuclein pre-formed fibril; PS1: Presenilin 1; PTX3: Pentraxin 3; SNpc: *Substantia nigra pars compacta*; S100β: S100 calcium-binding protein B; Tg: transgenic; TIMP-1: tissue inhibitor of metalloproteinase-1; TLR4: Toll-like receptor 4; TNFα: tumor necrosis factor α.

## 5. Conclusions

The diversity of astrocyte populations, each with its unique characteristics and functions, emphasizes the complex nature of their involvement in neurodegenerative diseases. Some astrocytes contribute to a pro-inflammatory environment by exacerbating neurodegeneration, whereas others exhibit neuroprotective functions. The dysregulation between neurotoxic and neuroprotective states is pivotal in disease pathogenesis. Therapeutic avenues targeting the suppression of neuroinflammatory astrocytes, or the enhancement of neuroprotective astrocytes hold great potential for treating these neurodegenerative disorders. Agents such as GLP-1RA, statins, or DMF can modulate astrocytes function, offering promising approaches. 

Further research is imperative to unravel intricate glial interactions, devise better astrocyte-targeting therapies, and evaluate their long-term safety and efficacy. Understanding the myriad morphologies and molecular pathways governing astrocyte activation may unveil novel therapeutic strategies. In sum, prioritizing the control of astrocyte reactivity is a promising avenue in the quest for innovative treatments against neurodegenerative diseases.

## Data Availability

The data that support the findings of this study are available from the corresponding author upon reasonable request.

## References

[1] Jakel S., Dimou L. (2017). Glial Cells and Their Function in the Adult Brain: A Journey through the History of Their Ablation. Front. Cell Neurosci..

[2] Colonna M., Butovsky O. (2017). Microglia Function in the Central Nervous System During Health and Neurodegeneration. Annu. Rev. Immunol..

[3] Barres B.A. (2008). The Mystery and Magic of Glia: A Perspective on Their Roles in Health and Disease. Neuron.

[4] Ding Z.B., Song L.J., Wang Q., Kumar G., Yan Y.Q., Ma C.G. (2021). Astrocytes: A Double-Edged Sword in Neurodegenerative Diseases. Neural Regen. Res..

[5] Ciurea A.V., Mohan A.G., Covache-Busuioc R.A., Costin H.P., Saceleanu V.M. (2023). The Brain’s Glymphatic System: Drawing New Perspectives in Neuroscience. Brain Sci..

[6] Cajal S.R. (1913). Contribución Al Conocimiento De La Neuroglia Del Cerebro Humano. Trab. Lab. Inv. Biol. Univ. Madrid.

[7] Zamanian J.L., Xu L., Foo L.C., Nouri N., Zhou L., Giffard R.G., Barres B.A. (2012). Genomic Analysis of Reactive Astrogliosis. J. Neurosci..

[8] Liddelow S.A., Guttenplan K.A., Clarke L.E., Bennett F.C., Bohlen C.J., Schirmer L., Bennett M.L., Munch A.E., Chung W.S., Peterson T.C. (2017). Neurotoxic Reactive Astrocytes Are Induced by Activated Microglia. Nature.

[9] Taylor X., Cisternas P., Jury N., Martinez P., Huang X., You Y., Redding-Ochoa J., Vidal R., Zhang J., Troncoso J. (2022). Activated Endothelial Cells Induce a Distinct Type of Astrocytic Reactivity. Commun. Biol..

[10] Liu L.R., Liu J.C., Bao J.S., Bai Q.Q., Wang G.Q. (2020). Interaction of Microglia and Astrocytes in the Neurovascular Unit. Front. Immunol..

[11] Sofroniew M.V. (2020). Astrocyte Reactivity: Subtypes, States, and Functions in Cns Innate Immunity. Trends Immunol..

[12] Escartin C., Galea E., Lakatos A., O’Callaghan J.P., Petzold G.C., Serrano-Pozo A., Steinhauser C., Volterra A., Carmignoto G., Agarwal A. (2021). Reactive Astrocyte Nomenclature, Definitions, and Future Directions. Nat. Neurosci..

[13] Palpagama T.H., Waldvogel H.J., Faull R.L.M., Kwakowsky A. (2019). The Role of Microglia and Astrocytes in Huntington’s Disease. Front. Mol. Neurosci..

[14] Liddelow S.A., Barres B.A. (2017). Reactive Astrocytes: Production, Function, and Therapeutic Potential. Immunity.

[15] Leitner G.R., Wenzel T.J., Marshall N., Gates E.J., Klegeris A. (2019). Targeting Toll-Like Receptor 4 to Modulate Neuroinflammation in Central Nervous System Disorders. Expert. Opin. Ther. Targets.

[16] Li L., Acioglu C., Heary R.F., Elkabes S. (2021). Role of Astroglial Toll-Like Receptors (Tlrs) in Central Nervous System Infections, Injury and Neurodegenerative Diseases. Brain Behav. Immun..

[17] Pascual O., Achour S.B., Rostaing P., Triller A., Bessis A. (2012). Microglia Activation Triggers Astrocyte-Mediated Modulation of Excitatory Neurotransmission. Proc. Natl. Acad. Sci. USA.

[18] Zhang L., Zhang J., Jiang X., Yang L., Zhang Q., Wang B., Cui L., Wang X. (2020). Hydroxytyrosol Inhibits Lps-Induced Neuroinflammatory Responses via Suppression of Tlr-4-Mediated Nf-κb P65 Activation and Erk Signaling Pathway. Neuroscience.

[19] Liu T., Zhang L., Joo D., Sun S.C. (2017). Nf-κb Signaling in Inflammation. Signal Transduct. Target. Ther..

[20] Ouali Alami N., Schurr C., Heuvel F.O., Tang L., Li Q., Tasdogan A., Kimbara A., Nettekoven M., Ottaviani G., Raposo C. (2018). Nf-κb Activation in Astrocytes Drives a Stage-Specific Beneficial Neuroimmunological Response in Als. EMBO J..

[21] Lattke M., Reichel S.N., Baumann B. (2017). Nf-κb-Mediated Astrocyte Dysfunction Initiates Neurodegeneration. Oncotarget.

[22] Li S., Fang Y., Zhang Y., Song M., Zhang X., Ding X., Yao H., Chen M., Sun Y., Ding J. (2022). Microglial Nlrp3 Inflammasome Activates Neurotoxic Astrocytes in Depression-Like Mice. Cell Rep..

[23] Xiao T., Ji H., Shangguan X., Qu S., Cui Y., Xu J. (2022). Nlrp3 Inflammasome of Microglia Promotes A1 Astrocyte Transformation, Neo-Neuron Decline and Cognition Impairment in Endotoxemia. Biochem. Biophys. Res. Commun..

[24] Wu P., Wu X., Zhou G., Wang Y., Liu X., Lv R., Liu Y., Wen Q. (2022). P2x7 Receptor-Induced Bone Cancer Pain by Regulating Microglial Activity via Nlrp3/Il-1beta Signaling. Pain. Physician.

[25] Surprenant A., Rassendren F., Kawashima E., North R.A., Buell G. (1996). The Cytolytic P2z Receptor for Extracellular Atp Identified as a P2x Receptor (P2x7). Science.

[26] Illes P. (2020). P2x7 Receptors Amplify Cns Damage in Neurodegenerative Diseases. Int. J. Mol. Sci..

[27] Cai R., Wang Y., Huang Z., Zou Q., Pu Y., Yu C., Cai Z. (2021). Role of Rhoa/Rock Signaling in Alzheimer’s Disease. Behav. Brain Res..

[28] Soto-Rojas L.O., Martinez-Davila I.A., Luna-Herrera C., Gutierrez-Castillo M.E., Lopez-Salas F.E., Gatica-Garcia B., Soto-Rodriguez G., Tobon M.E.B., Flores G., Padilla-Viveros A. (2020). Unilateral Intranigral Administration of Beta-Sitosterol Beta-D-Glucoside Triggers Pathological Alpha-Synuclein Spreading and Bilateral Nigrostriatal Dopaminergic Neurodegeneration in the Rat. Acta Neuropathol. Commun..

[29] Lu W., Wen J., Chen Z. (2020). Distinct Roles of Rock1 and Rock2 on the Cerebral Ischemia Injury and Subsequently Neurodegenerative Changes. Pharmacology.

[30] Das Sarma J., Ciric B., Marek R., Sadhukhan S., Caruso M.L., Shafagh J., Fitzgerald D.C., Shindler K.S., Rostami A. (2009). Functional Interleukin-17 Receptor a Is Expressed in Central Nervous System Glia and Upregulated in Experimental Autoimmune Encephalomyelitis. J. Neuroinflamm..

[31] Luo H., Liu H.Z., Zhang W.W., Matsuda M., Lv N., Chen G., Xu Z.Z., Zhang Y.Q. (2019). Interleukin-17 Regulates Neuron-Glial Communications, Synaptic Transmission, and Neuropathic Pain after Chemotherapy. Cell Rep..

[32] Kohler S., Winkler U., Junge T., Lippmann K., Eilers J., Hirrlinger J. (2023). Gray and White Matter Astrocytes Differ in Basal Metabolism but Respond Similarly to Neuronal Activity. Glia.

[33] Ceyzeriat K., Abjean L., Sauvage M.A.C.-D., Haim L.B., Escartin C. (2016). The Complex States of Astrocyte Reactivity: How Are They Controlled by the Jak-Stat3 Pathway?. Neuroscience.

[34] Wang T., Yuan W., Liu Y., Zhang Y., Wang Z., Zhou X., Ning G., Zhang L., Yao L., Feng S. (2015). The Role of the Jak-Stat Pathway in Neural Stem Cells, Neural Progenitor Cells and Reactive Astrocytes after Spinal Cord Injury. Biomed. Rep..

[35] Wang Y., van Boxel-Dezaire A.H., Cheon H., Yang J., Stark G.R. (2013). Stat3 Activation in Response to Il-6 Is Prolonged by the Binding of Il-6 Receptor to Egf Receptor. Proc. Natl. Acad. Sci. USA.

[36] McGeachy M.J., Bak-Jensen K.S., Chen Y., Tato C.M., Blumenschein W., McClanahan T., Cua D.J. (2007). Tgf-Beta and Il-6 Drive the Production of Il-17 and Il-10 by T Cells and Restrain T(H)-17 Cell-Mediated Pathology. Nat. Immunol..

[37] Zhou Z., Peng X., Insolera R., Fink D.J., Mata M. (2009). Il-10 Promotes Neuronal Survival Following Spinal Cord Injury. Exp. Neurol..

[38] Gupta S., Mishra K., Surolia A., Banerjee K. (2011). Suppressor of Cytokine Signalling-6 Promotes Neurite Outgrowth via Jak2/Stat5-Mediated Signalling Pathway, Involving Negative Feedback Inhibition. PLoS ONE.

[39] Zhu Y., Liu Z., Peng Y.P., Qiu Y.H. (2017). Interleukin-10 Inhibits Neuroinflammation-Mediated Apoptosis of Ventral Mesencephalic Neurons via Jak-Stat3 Pathway. Int. Immunopharmacol..

[40] Wang J., Sareddy G.R., Lu Y., Pratap U.P., Tang F., Greene K.M., Meyre P.L., Tekmal R.R., Vadlamudi R.K., Brann D.W. (2020). Astrocyte-Derived Estrogen Regulates Reactive Astrogliosis and Is Neuroprotective Following Ischemic Brain Injury. J. Neurosci..

[41] Pang Q.M., Zhang Q., Wu X.C., Yang R.L., Fu S.P., Fan Z.H., Liu J., Yu L.M., Peng J.C., Zhang T. (2023). Mechanism of M2 Macrophages Modulating Astrocyte Polarization through the Tgf-Beta/Pi3k/Akt Pathway. Immunol. Lett..

[42] Goyal A., Agrawal A., Verma A., Dubey N. (2023). The Pi3k-Akt Pathway: A Plausible Therapeutic Target in Parkinson’s Disease. Exp. Mol. Pathol..

[43] Li T., Liu T., Chen X., Li L., Feng M., Zhang Y., Wan L., Zhang C., Yao W. (2020). Microglia Induce the Transformation of A1/A2 Reactive Astrocytes via the Cxcr7/Pi3k/Akt Pathway in Chronic Post-Surgical Pain. J. Neuroinflamm..

[44] Liu R., Wang W., Wang S., Xie W., Li H., Ning B. (2018). Microrna-21 Regulates Astrocytic Reaction Post-Acute Phase of Spinal Cord Injury through Modulating Tgf-Beta Signaling. Aging.

[45] Song G., Yang R., Zhang Q., Chen L., Huang D., Zeng J., Yang C., Zhang T. (2019). Tgf-Beta Secretion by M2 Macrophages Induces Glial Scar Formation by Activating Astrocytes In Vitro. J. Mol. Neurosci..

[46] Xu X., Zhang A., Zhu Y., He W., Di W., Fang Y., Shi X. (2018). Mfg-E8 Reverses Microglial-Induced Neurotoxic Astrocyte (A1) via Nf-κb and Pi3k-Akt Pathways. J. Cell Physiol..

[47] Cekanaviciute E., Dietrich H.K., Axtell R.C., Williams A.M., Egusquiza R., Wai K.M., Koshy A.A., Buckwalter M.S. (2014). Astrocytic Tgf-Beta Signaling Limits Inflammation and Reduces Neuronal Damage during Central Nervous System Toxoplasma Infection. J. Immunol..

[48] Divolis G., Stavropoulos A., Manioudaki M., Apostolidou A., Doulou A., Gavriil A., Dafnis I., Chroni A., Mummery C., Xilouri M. (2019). Activation of Both Transforming Growth Factor-Beta and Bone Morphogenetic Protein Signalling Pathways Upon Traumatic Brain Injury Restrains Pro-Inflammatory and Boosts Tissue Reparatory Responses of Reactive Astrocytes and Microglia. Brain Commun..

[49] Feng X.F., Li M.C., Lin Z.Y., Li M.Z., Lu Y., Zhuang Y.M., Lei J.F., Wang L., Zhao H. (2023). Tetramethylpyrazine Promotes Stroke Recovery by Inducing the Restoration of Neurovascular Unit and Transformation of A1/A2 Reactive Astrocytes. Front. Cell Neurosci..

[50] Westergard T., Rothstein J.D. (2020). Astrocyte Diversity: Current Insights and Future Directions. Neurochem. Res..

[51] Zhou B., Zuo Y.X., Jiang R.T. (2019). Astrocyte Morphology: Diversity, Plasticity, and Role in Neurological Diseases. CNS Neurosci. Ther..

[52] Jakobs T.C., Bakota L., Brandt R. (2014). Analysis of Morphology and Structural Remodeling of Astrocytes. Laser Scanning Microscopy and Quantitative Image Analysis of Neuronal Tissue.

[53] Sun D., Lye-Barthel M., Masland R.H., Jakobs T.C. (2010). Structural Remodeling of Fibrous Astrocytes after Axonal Injury. J. Neurosci..

[54] Torres-Ceja B., Olsen M.L. (2022). A Closer Look at Astrocyte Morphology: Development, Heterogeneity, and Plasticity at Astrocyte Leaflets. Curr. Opin. Neurobiol..

[55] Khakh B.S., Deneen B. (2019). The Emerging Nature of Astrocyte Diversity. Annu. Rev. Neurosci..

[56] Stogsdill J.A., Ramirez J., Liu D., Kim Y.H., Baldwin K.T., Enustun E., Ejikeme T., Ji R.R., Eroglu C. (2017). Astrocytic Neuroligins Control Astrocyte Morphogenesis and Synaptogenesis. Nature.

[57] Acaz-Fonseca E., Ortiz-Rodriguez A., Azcoitia I., Garcia-Segura L.M., Arevalo M.A. (2019). Notch Signaling in Astrocytes Mediates Their Morphological Response to an Inflammatory Challenge. Cell Death Discov..

[58] Jurga A.M., Paleczna M., Kadluczka J., Kuter K.Z. (2021). Beyond the Gfap-Astrocyte Protein Markers in the Brain. Biomolecules.

[59] Fan Y.Y., Huo J. (2021). A1/A2 Astrocytes in Central Nervous System Injuries and Diseases: Angels or Devils?. Neurochem. Int..

[60] Zou L.H., Shi Y.J., He H., Jiang S.M., Huo F.F., Wang X.M., Wu F., Ma L. (2019). Effects of Fgf2/Fgfr1 Pathway on Expression of A1 Astrocytes after Infrasound Exposure. Front. Neurosci..

[61] Althammer F., Ferreira-Neto H.C., Rubaharan M., Roy R.K., Patel A.A., Murphy A., Cox D.N., Stern J.E. (2020). Correction To: Three-Dimensional Morphometric Analysisreveals Time-Dependent Structural Changesin Microglia and Astrocytes in the Centralamygdala and Hypothalamicparaventricular Nucleus of Heart Failure Rats. J. Neuroinflamm..

[62] Clark D.P.Q., Perreau V.M., Shultz S.R., Brady R.D., Lei E., Dixit S., Taylor J.M., Beart P.M., Boon W.C. (2019). Inflammation in Traumatic Brain Injury: Roles for Toxic A1 Astrocytes and Microglial-Astrocytic Crosstalk. Neurochem. Res..

[63] Przedborski S., Vila M., Jackson-Lewis V. (2003). Neurodegeneration: What Is It and Where Are We?. J. Clin. Investig..

[64] McColgan P., Joubert J., Tabrizi S.J., Rees G. (2020). The Human Motor Cortex Microcircuit: Insights for Neurodegenerative Disease. Nat. Rev. Neurosci..

[65] Dugger B.N., Dickson D.W. (2017). Pathology of Neurodegenerative Diseases. Cold Spring Harb. Perspect. Biol..

[66] Jorgačevski J., Potokar M. (2023). Immune Functions of Astrocytes in Viral Neuroinfections. Int. J. Mol. Sci..

[67] Elsherbini D.M.A., Ghoneim F.M., El-Mancy E.M., Ebrahim H.A., El-Sherbiny M., El-Shafey M., Al-Serwi R.H., Elsherbiny N.M. (2022). Astrocytes Profiling in Acute Hepatic Encephalopathy: Possible Enrolling of Glial Fibrillary Acidic Protein, Tumor Necrosis Factor-Alpha, Inwardly Rectifying Potassium Channel (Kir 4.1) and Aquaporin-4 in Rat Cerebral Cortex. Front. Cell Neurosci..

[68] Kruyer A., Kalivas P.W., Scofield M.D. (2023). Astrocyte Regulation of Synaptic Signaling in Psychiatric Disorders. Neuropsychopharmacology.

[69] Shen X.Y., Gao Z.K., Han Y., Yuan M., Guo Y.S., Bi X. (2021). Activation and Role of Astrocytes in Ischemic Stroke. Front. Cell Neurosci..

[70] Yuan M., Wu H. (2022). Astrocytes in the Traumatic Brain Injury: The Good and the Bad. Exp. Neurol..

[71] Barbar L., Jain T., Zimmer M., Kruglikov I., Sadick J.S., Wang M., Kalpana K., Rose I.V.L., Burstein S.R., Rusielewicz T. (2020). Cd49f Is a Novel Marker of Functional and Reactive Human Ipsc-Derived Astrocytes. Neuron.

[72] Mei L., Nave K.A. (2014). Neuregulin-Erbb Signaling in the Nervous System and Neuropsychiatric Diseases. Neuron.

[73] de Mezer M., Rogaliński J., Przewoźny S., Chojnicki M., Niepolski L., Sobieska M., Przystańska A. (2023). Serpina3, Stimulator or Inhibitor of Pathological Changes. Biomedicines.

[74] Kim H., Leng K., Park J., Sorets A.G., Kim S., Shostak A., Embalabala R.J., Mlouk K., Katdare K.A., Rose I.V.L. (2022). Reactive Astrocytes Transduce Inflammation in a Blood-Brain Barrier Model through a Tnf-Stat3 Signaling Axis and Secretion of Alpha 1-Antichymotrypsin. Nat. Commun..

[75] Norton E.S., Da Mesquita S., Guerrero-Cazares H. (2021). Serpina3 in Glioblastoma and Alzheimer’s Disease. Aging.

[76] Dejanovic B., Wu T., Tsai M.-C., Graykowski D., Gandham V.D., Rose C.M., Bakalarski C.E., Ngu H., Wang Y., Pandey S. (2022). Complement C1q-Dependent Excitatory and Inhibitory Synapse Elimination by Astrocytes and Microglia in Alzheimer’s Disease Mouse Models. Nat. Aging.

[77] Gao Z., Zhang C., Feng Z., Liu Z., Yang Y., Yang K., Chen L., Yao R. (2022). C1q Inhibits Differentiation of Oligodendrocyte Progenitor Cells via Wnt/Beta-Catenin Signaling Activation in a Cuprizone-Induced Mouse Model of Multiple Sclerosis. Exp. Neurol..

[78] Lian H., Litvinchuk A., Chiang A.C.-A., Aithmitti N., Jankowsky J.L., Zheng H. (2016). Astrocyte-Microglia Cross Talk through Complement Activation Modulates Amyloid Pathology in Mouse Models of Alzheimer’s Disease. J. Neurosci..

[79] Lian H., Yang L., Cole A., Sun L., Chiang A.C., Fowler S.W., Shim D.J., Rodriguez-Rivera J., Taglialatela G., Jankowsky J.L. (2015). Nfκb-Activated Astroglial Release of Complement C3 Compromises Neuronal Morphology and Function Associated with Alzheimer’s Disease. Neuron.

[80] Linnerbauer M., Wheeler M.A., Quintana F.J. (2020). Astrocyte Crosstalk in Cns Inflammation. Neuron.

[81] Dikmen H.O., Hemmerich M., Lewen A., Hollnagel J.O., Chausse B., Kann O. (2020). Gm-Csf Induces Noninflammatory Proliferation of Microglia and Disturbs Electrical Neuronal Network Rhythms In Situ. J. Neuroinflamm..

[82] Lotfi N., Thome R., Rezaei N., Zhang G.X., Rezaei A., Rostami A., Esmaeil N. (2019). Roles of Gm-Csf in the Pathogenesis of Autoimmune Diseases: An Update. Front. Immunol..

[83] Lawrence J.M., Schardien K., Wigdahl B., Nonnemacher M.R. (2023). Roles of Neuropathology-Associated Reactive Astrocytes: A Systematic Review. Acta Neuropathol. Commun..

[84] Rothhammer V., Kenison J.E., Tjon E., Takenaka M.C., de Lima K.A., Borucki D.M., Chao C.C., Wilz A., Blain M., Healy L. (2017). Sphingosine 1-Phosphate Receptor Modulation Suppresses Pathogenic Astrocyte Activation and Chronic Progressive Cns Inflammation. Proc. Natl. Acad. Sci. USA.

[85] Gutiérrez I.L., Novellino F., Caso J.R., García-Bueno B., Leza J.C., Madrigal J.L.M. (2022). CCL2 Inhibition of Pro-Resolving Mediators Potentiates Neuroinflammation in Astrocytes. Int. J. Mol. Sci..

[86] He M., Dong H., Huang Y., Lu S., Zhang S., Qian Y., Jin W. (2016). Astrocyte-Derived Ccl2 Is Associated with M1 Activation and Recruitment of Cultured Microglial Cells. Cell Physiol. Biochem..

[87] Kim R.Y., Hoffman A.S., Itoh N., Ao Y., Spence R., Sofroniew M.V., Voskuhl R.R. (2014). Astrocyte Ccl2 Sustains Immune Cell Infiltration in Chronic Experimental Autoimmune Encephalomyelitis. J. Neuroimmunol..

[88] Zhou Z., Lin T., Liu Z., Ding Q., Ma Z., Li W., Xie F., Lan Y., Feng Y. (2022). Il-17a Mediates Demyelination by Activating A1 Astrocytes via Socs3 During Angiostrongylus Cantonensis Infection. Front. Immunol..

[89] Qian Y., Liu C., Hartupee J., Altuntas C.Z., Gulen M.F., Jane-Wit D., Xiao J., Lu Y., Giltiay N., Liu J. (2007). The Adaptor Act1 Is Required for Interleukin 17-Dependent Signaling Associated with Autoimmune and Inflammatory Disease. Nat. Immunol..

[90] Fernandez-Garcia S., Sancho-Balsells A., Longueville S., Herve D., Gruart A., Delgado-Garcia J.M., Alberch J., Giralt A. (2020). Astrocytic Bdnf and Trkb Regulate Severity and Neuronal Activity in Mouse Models of Temporal Lobe Epilepsy. Cell Death Dis..

[91] Colombo E., Cordiglieri C., Melli G., Newcombe J., Krumbholz M., Parada L.F., Medico E., Hohlfeld R., Meinl E., Farina C. (2012). Stimulation of the Neurotrophin Receptor Trkb on Astrocytes Drives Nitric Oxide Production and Neurodegeneration. J. Exp. Med..

[92] Valenzuela-Arzeta I.E., Soto-Rojas L.O., Flores-Martinez Y.M., Delgado-Minjares K.M., Gatica-Garcia B., Mascotte-Cruz J.U., Nava P., Aparicio-Trejo O.E., Reyes-Corona D., Martinez-Davila I.A. (2023). Lps Triggers Acute Neuroinflammation and Parkinsonism Involving Nlrp3 Inflammasome Pathway and Mitochondrial Ci Dysfunction in the Rat. Int. J. Mol. Sci..

[93] She N., Shi Y., Feng Y., Ma L., Yuan Y., Zhang Y., Cao Z., Chen X., Zhao B., Liu H. (2022). Nlrp3 Inflammasome Regulates Astrocyte Transformation in Brain Injury Induced by Chronic Intermittent Hypoxia. BMC Neurosci..

[94] Johann S., Heitzer M., Kanagaratnam M., Goswami A., Rizo T., Weis J., Troost D., Beyer C. (2015). Nlrp3 Inflammasome Is Expressed by Astrocytes in the Sod1 Mouse Model of Als and in Human Sporadic Als Patients. Glia.

[95] Guttenplan K.A., Weigel M.K., Prakash P., Wijewardhane P.R., Hasel P., Rufen-Blanchette U., Munch A.E., Blum J.A., Fine J., Neal M.C. (2021). Neurotoxic Reactive Astrocytes Induce Cell Death via Saturated Lipids. Nature.

[96] Killoy K.M., Harlan B.A., Pehar M., Vargas M.R. (2020). Fabp7 Upregulation Induces a Neurotoxic Phenotype in Astrocytes. Glia.

[97] Qian D., Li L., Rong Y., Liu W., Wang Q., Zhou Z., Gu C., Huang Y., Zhao X., Chen J. (2019). Blocking Notch Signal Pathway Suppresses the Activation of Neurotoxic A1 Astrocytes after Spinal Cord Injury. Cell Cycle.

[98] Li S., Uno Y., Rudolph U., Cobb J., Liu J., Anderson T., Levy D., Balu D.T., Coyle J.T. (2018). Astrocytes in Primary Cultures Express Serine Racemase, Synthesize D-Serine and Acquire A1 Reactive Astrocyte Features. Biochem. Pharmacol..

[99] Shi J., Xu H., Cavagnaro M.J., Li X., Fang J. (2021). Blocking Hmgb1/Rage Signaling by Berberine Alleviates A1 Astrocyte and Attenuates Sepsis-Associated Encephalopathy. Front. Pharmacol..

[100] Song N., Zhu H., Xu R., Liu J., Fang Y., Zhang J., Ding J., Hu G., Lu M. (2023). Corrigendum: Induced Expression of Kir6.2 in A1 Astrocytes Propagates Inflammatory Neurodegeneration via Drp1-Dependent Mitochondrial Fission. Front. Pharmacol..

[101] Vismara I., Papa S., Veneruso V., Mauri E., Mariani A., De Paola M., Affatato R., Rossetti A., Sponchioni M., Moscatelli D. (2020). Selective Modulation of A1 Astrocytes by Drug-Loaded Nano-Structured Gel in Spinal Cord Injury. ACS Nano.

[102] Rathore K.I., Berard J.L., Redensek A., Chierzi S., Lopez-Vales R., Santos M., Akira S., David S. (2011). Lipocalin 2 Plays an Immunomodulatory Role and Has Detrimental Effects after Spinal Cord Injury. J. Neurosci..

[103] L’Episcopo F., Tirolo C., Testa N., Caniglia S., Morale M.C., Cossetti C., D’Adamo P., Zardini E., Andreoni L., Ihekwaba A.E. (2011). Reactive Astrocytes and Wnt/Beta-Catenin Signaling Link Nigrostriatal Injury to Repair in 1-Methyl-4-Phenyl-1,2,3,6-Tetrahydropyridine Model of Parkinson’s Disease. Neurobiol. Dis..

[104] Chacon M.A., Varela-Nallar L., Inestrosa N.C. (2008). Frizzled-1 Is Involved in the Neuroprotective Effect of Wnt3a against Abeta Oligomers. J. Cell Physiol..

[105] Ju Y.H., Bhalla M., Hyeon S.J., Oh J.E., Yoo S., Chae U., Kwon J., Koh W., Lim J., Park Y.M. (2022). Astrocytic Urea Cycle Detoxifies Abeta-Derived Ammonia While Impairing Memory in Alzheimer’s Disease. Cell Metab..

[106] Dinkova-Kostova A.T., Kostov R.V., Kazantsev A.G. (2018). The Role of Nrf2 Signaling in Counteracting Neurodegenerative Diseases. FEBS J..

[107] Gupta K., Patani R., Baxter P., Serio A., Story D., Tsujita T., Hayes J.D., Pedersen R.A., Hardingham G.E., Chandran S. (2012). Human Embryonic Stem Cell Derived Astrocytes Mediate Non-Cell-Autonomous Neuroprotection through Endogenous and Drug-Induced Mechanisms. Cell Death Differ..

[108] Vargas M.R., Johnson D.A., Sirkis D.W., Messing A., Johnson J.A. (2008). Nrf2 Activation in Astrocytes Protects against Neurodegeneration in Mouse Models of Familial Amyotrophic Lateral Sclerosis. J. Neurosci..

[109] Lee J.Y., Han S.H., Park M.H., Baek B., Song I.S., Choi M.K., Takuwa Y., Ryu H., Kim S.H., He X. (2018). Neuronal Sphk1 Acetylates Cox2 and Contributes to Pathogenesis in a Model of Alzheimer’s Disease. Nat. Commun..

[110] Zhang X., Shen Z.-L., Ji Y.-W., Yin C., Xiao C., Zhou C. (2023). Activation and Polarization of Striatal Microglia and Astrocytes Are Involved in Bradykinesia and Allodynia in Early-Stage Parkinsonian Mice. Fundam. Res..

[111] Dong X., Zhang Z., Shu X., Zhuang Z., Liu P., Liu R., Xia S., Bao X., Xu Y., Chen Y. (2023). Mfg-E8 Alleviates Cognitive Impairments Induced by Chronic Cerebral Hypoperfusion by Phagocytosing Myelin Debris and Promoting Remyelination. Neurosci. Bull..

[112] Cheyuo C., Aziz M., Wang P. (2019). Neurogenesis in Neurodegenerative Diseases: Role of Mfg-E8. Front. Neurosci..

[113] Lim Y.Y., Maruff P., Barthélemy N.R., Goate A., Hassenstab J., Sato C., Fagan A.M., Benzinger T.L., Xiong C., Cruchaga C. (2022). Association of Bdnf Val66met with Tau Hyperphosphorylation and Cognition in Dominantly Inherited Alzheimer Disease. JAMA Neurol..

[114] de Pins B., Cifuentes-Diaz C., Farah A.T., Lopez-Molina L., Montalban E., Sancho-Balsells A., Lopez A., Gines S., Delgado-Garcia J.M., Alberch J. (2019). Conditional Bdnf Delivery from Astrocytes Rescues Memory Deficits, Spine Density, and Synaptic Properties in the 5xfad Mouse Model of Alzheimer Disease. J. Neurosci..

[115] Sidoryk-Wegrzynowicz M., Gerber Y.N., Ries M., Sastre M., Tolkovsky A.M., Spillantini M.G. (2017). Astrocytes in Mouse Models of Tauopathies Acquire Early Deficits and Lose Neurosupportive Functions. Acta Neuropathol. Commun..

[116] Doyle K.P., Cekanaviciute E., Mamer L.E., Buckwalter M.S. (2010). Tgfbeta Signaling in the Brain Increases with Aging and Signals to Astrocytes and Innate Immune Cells in the Weeks after Stroke. J. Neuroinflamm..

[117] Grammas P., Ovase R. (2002). Cerebrovascular Transforming Growth Factor-Beta Contributes to Inflammation in the Alzheimer’s Disease Brain. Am. J. Pathol..

[118] Li X., Feng X., Sun X., Hou N., Han F., Liu Y. (2022). Global, Regional, and National Burden of Alzheimer’s Disease and Other Dementias, 1990–2019. Front Aging Neurosci.

[119] Maccioni R.B., Tapia J.P., Guzman-Martinez L. (2018). Pathway to Tau Modifications and the Origins of Alzheimer’s Disease. Arch. Med. Res..

[120] Podlesny-Drabiniok A., Marcora E., Goate A.M. (2020). Microglial Phagocytosis: A Disease-Associated Process Emerging from Alzheimer’s Disease Genetics. Trends Neurosci..

[121] Serrano-Pozo A., Muzikansky A., Gomez-Isla T., Growdon J.H., Betensky R.A., Frosch M.P., Hyman B.T. (2013). Differential Relationships of Reactive Astrocytes and Microglia to Fibrillar Amyloid Deposits in Alzheimer Disease. J. Neuropathol. Exp. Neurol..

[122] Bellaver B., Povala G., Ferreira P.C.L., Ferrari-Souza J.P., Leffa D.T., Lussier F.Z., Benedet A.L., Ashton N.J., Triana-Baltzer G., Kolb H.C. (2023). Astrocyte Reactivity Influences Amyloid-Beta Effects on Tau Pathology in Preclinical Alzheimer’s Disease. Nat. Med..

[123] Li S., Jin M., Koeglsperger T., Shepardson N.E., Shankar G.M., Selkoe D.J. (2011). Soluble Abeta Oligomers Inhibit Long-Term Potentiation through a Mechanism Involving Excessive Activation of Extrasynaptic Nr2b-Containing Nmda Receptors. J. Neurosci..

[124] Li K., Liu S., Yao S., Wang B., Dai D., Yao L. (2009). Interaction between Interleukin-8 and Methylenetetrahydrofolate Reductase Genes Modulates Alzheimer’s Disease Risk. Dement. Geriatr. Cogn. Disord..

[125] Delekate A., Fuchtemeier M., Schumacher T., Ulbrich C., Foddis M., Petzold G.C. (2014). Metabotropic P2y1 Receptor Signalling Mediates Astrocytic Hyperactivity in Vivo in an Alzheimer’s Disease Mouse Model. Nat. Commun..

[126] Olabarria M., Noristani H.N., Verkhratsky A., Rodriguez J.J. (2010). Concomitant Astroglial Atrophy and Astrogliosis in a Triple Transgenic Animal Model of Alzheimer’s Disease. Glia.

[127] Burbach G.J., Dehn D., Del Turco D., Staufenbiel M., Deller T. (2004). Laser Microdissection Reveals Regional and Cellular Differences in Gfap Mrna Upregulation Following Brain Injury, Axonal Denervation, and Amyloid Plaque Deposition. Glia.

[128] Habib N., McCabe C., Medina S., Varshavsky M., Kitsberg D., Dvir-Szternfeld R., Green G., Dionne D., Nguyen L., Marshall J.L. (2020). Disease-Associated Astrocytes in Alzheimer’s Disease and Aging. Nat. Neurosci..

[129] Yun S.P., Kam T.I., Panicker N., Kim S., Oh Y., Park J.S., Kwon S.H., Park Y.J., Karuppagounder S.S., Park H. (2018). Block of A1 Astrocyte Conversion by Microglia Is Neuroprotective in Models of Parkinson’s Disease. Nat. Med..

[130] Park J.S., Kam T.I., Lee S., Park H., Oh Y., Kwon S.H., Song J.J., Kim D., Kim H., Jhaldiyal A. (2021). Blocking Microglial Activation of Reactive Astrocytes Is Neuroprotective in Models of Alzheimer’s Disease. Acta Neuropathol. Commun..

[131] Zhang M., Wu Y., Gao R., Chen X., Chen R., Chen Z. (2022). Glucagon-Like Peptide-1 Analogs Mitigate Neuroinflammation in Alzheimer’s Disease by Suppressing Nlrp2 Activation in Astrocytes. Mol. Cell Endocrinol..

[132] Xie Y., Zheng J., Li S., Li H., Zhou Y., Zheng W., Zhang M., Liu L., Chen Z. (2021). Glp-1 Improves the Neuronal Supportive Ability of Astrocytes in Alzheimer’s Disease by Regulating Mitochondrial Dysfunction via the Camp/Pka Pathway. Biochem. Pharmacol..

[133] Gharagozloo M., Smith M.D., Sotirchos E.S., Jin J., Meyers K., Taylor M., Garton T., Bannon R., Lord H.N., Dawson T.M. (2021). Therapeutic Potential of a Novel Glucagon-Like Peptide-1 Receptor Agonist, Nly01, in Experimental Autoimmune Encephalomyelitis. Neurotherapeutics.

[134] Sterling J.K., Adetunji M.O., Guttha S., Bargoud A.R., Uyhazi K.E., Ross A.G., Dunaief J.L., Cui Q.N. (2020). Glp-1 Receptor Agonist Nly01 Reduces Retinal Inflammation and Neuron Death Secondary to Ocular Hypertension. Cell Rep..

[135] Darvish Khadem M., Tabandeh M.R., Haschemi A., Kheirollah A., Shahriari A. (2022). Dimethyl Itaconate Reprograms Neurotoxic to Neuroprotective Primary Astrocytes through the Regulation of Nlrp3 Inflammasome and Nrf2/Ho-1 Pathways. Mol. Cell Neurosci..

[136] Guo M.F., Zhang H.Y., Li Y.H., Gu Q.F., Wei W.Y., Wang Y.Y., Zhang X.J., Liu X.Q., Song L.J., Chai Z. (2020). Fasudil Inhibits the Activation of Microglia and Astrocytes of Transgenic Alzheimer’s Disease Mice via the Downregulation of Tlr4/Myd88/Nf-κb Pathway. J. Neuroimmunol..

[137] Sarkar S., Biswas S.C. (2021). Astrocyte Subtype-Specific Approach to Alzheimer’s Disease Treatment. Neurochem. Int..

[138] Shi J.Z., Zheng X.M., Zhou Y.F., Yun L.Y., Luo D.M., Hao J.J., Liu P.F., Zhang W.K., Xu J.K., Yan Y. (2022). Cornuside Is a Potential Agent against Alzheimer’s Disease via Orchestration of Reactive Astrocytes. Nutrients.

[139] Saha P., Sarkar S., Paidi R.K., Biswas S.C. (2020). Timp-1, A Key Cytokine Released from Activated Astrocytes Protects Neurons and Ameliorates Cognitive Behaviours in a Rodent Model of Alzheimer’s Disease. Brain Behav. Immun..

[140] Dorsey E.R., Sherer T., Okun M.S., Bloem B.R. (2018). The Emerging Evidence of the Parkinson Pandemic. J. Park. Dis..

[141] Graves N.J., Gambin Y., Sierecki E. (2023). Alpha-Synuclein Strains and Their Relevance to Parkinson’s Disease, Multiple System Atrophy, and Dementia with Lewy Bodies. Int. J. Mol. Sci..

[142] Poewe W., Seppi K., Tanner C.M., Halliday G.M., Brundin P., Volkmann J., Schrag A.E., Lang A.E. (2017). Parkinson Disease. Nat. Rev. Dis. Primers.

[143] Rostami J., Mothes T., Kolahdouzan M., Eriksson O., Moslem M., Bergstrom J., Ingelsson M., O’Callaghan P., Healy L.M., Falk A. (2021). Crosstalk between Astrocytes and Microglia Results in Increased Degradation of Alpha-Synuclein and Amyloid-Beta Aggregates. J. Neuroinflamm..

[144] Morales-Martinez A., Martinez-Gomez P.A., Martinez-Fong D., Villegas-Rojas M.M., Perez-Severiano F., Del Toro-Colin M.A., Delgado-Minjares K.M., Blanco-Alvarez V.M., Leon-Chavez B.A., Aparicio-Trejo O.E. (2022). Oxidative Stress and Mitochondrial Complex I Dysfunction Correlate with Neurodegeneration in an Alpha-Synucleinopathy Animal Model. Int. J. Mol. Sci..

[145] Delgado-Minjares K.M., Martinez-Fong D., Martinez-Davila I.A., Banuelos C., Gutierrez-Castillo M.E., Blanco-Alvarez V.M., Cardenas-Aguayo M.D., Luna-Munoz J., Pacheco-Herrero M., Soto-Rojas L.O. (2021). Mechanistic Insight from Preclinical Models of Parkinson’s Disease Could Help Redirect Clinical Trial Efforts in Gdnf Therapy. Int. J. Mol. Sci..

[146] Iizumi T., Takahashi S., Mashima K., Minami K., Izawa Y., Abe T., Hishiki T., Suematsu M., Kajimura M., Suzuki N. (2016). A Possible Role of Microglia-Derived Nitric Oxide by Lipopolysaccharide in Activation of Astroglial Pentose-Phosphate Pathway via the Keap1/Nrf2 System. J. Neuroinflamm..

[147] Lee Y., Lee S., Chang S.C., Lee J. (2019). Significant Roles of Neuroinflammation in Parkinson’s Disease: Therapeutic Targets for Pd Prevention. Arch. Pharm. Res..

[148] Luna-Herrera C., Martinez-Davila I.A., Soto-Rojas L.O., Flores-Martinez Y.M., Fernandez-Parrilla M.A., Ayala-Davila J., Leon-Chavez B.A., Soto-Rodriguez G., Blanco-Alvarez V.M., Lopez-Salas F.E. (2020). Intranigral Administration of Beta-Sitosterol-Beta-D-Glucoside Elicits Neurotoxic A1 Astrocyte Reactivity and Chronic Neuroinflammation in the Rat Substantia Nigra. J. Immunol. Res..

[149] Kim M.L., Sung K.R., Kwon J., Choi G.W., Shin J.A. (2021). Neuroprotective Effect of Statins in a Rat Model of Chronic Ocular Hypertension. Int. J. Mol. Sci..

[150] Jafari M., Hojati V., Khaksari M., Vaezi G. (2021). Simvastatin Attenuates Spatial Memory Impairment via Inhibiting Microgliosis and Apoptotic Cell Death against Ethanol Induced Neurotoxicity in the Developing Rat Hippocampus. Brain Res..

[151] Willems S., Marschner J.A., Kilu W., Faudone G., Busch R., Duensing-Kropp S., Heering J., Merk D. (2022). Nurr1 Modulation Mediates Neuroprotective Effects of Statins. Adv. Sci..

[152] Popichak K.A., Hammond S.L., Moreno J.A., Afzali M.F., Backos D.S., Slayden R.D., Safe S., Tjalkens R.B. (2018). Compensatory Expression of Nur77 and Nurr1 Regulates Nf-κb-Dependent Inflammatory Signaling in Astrocytes. Mol. Pharmacol..

[153] Wang T., Cao X.B., Chen X.W., Huang P.P., Zhang T., Chen Z.B., Tang B.S. (2015). Influence of Simvastatin on Dopaminergic Neurons of Lipopolysaccharide-Induced Rat Model of Parkinson’s Disease. Asian Pac. J. Trop. Med..

[154] Carroll C.B., Webb D., Stevens K.N., Vickery J., Eyre V., Ball S., Wyse R., Webber M., Foggo A., Zajicek J. (2019). Simvastatin as a Neuroprotective Treatment for Parkinson’s Disease (Pd Stat): Protocol for a Double-Blind, Randomised, Placebo-Controlled Futility Study. BMJ Open.

[155] Stoker T.B., Mason S.L., Greenland J.C., Holden S.T., Santini H., Barker R.A. (2022). Huntington’s Disease: Diagnosis and Management. Pract. Neurol..

[156] Ghosh R., Tabrizi S.J. (2018). Clinical Features of Huntington’s Disease. Advances in Experimental Medicine and Biology.

[157] Diaz-Castro B., Gangwani M.R., Yu X., Coppola G., Khakh B.S. (2019). Astrocyte Molecular Signatures in Huntington’s Disease. Sci. Transl. Med..

[158] Yang H.M., Yang S., Huang S.S., Tang B.S., Guo J.F. (2017). Microglial Activation in the Pathogenesis of Huntington’s Disease. Front. Aging Neurosci..

[159] Jansen A.H., van Hal M., den Kelder I.C.O., Meier R.T., de Ruiter A.A., Schut M.H., Smith D.L., Grit C., Brouwer N., Kamphuis W. (2017). Frequency of Nuclear Mutant Huntingtin Inclusion Formation in Neurons and Glia Is Cell-Type-Specific. Glia.

[160] Saba J., Couselo F.L., Bruno J., Carniglia L., Durand D., Lasaga M., Caruso C. (2022). Neuroinflammation in Huntington’s Disease: A Starring Role for Astrocyte and Microglia. Curr. Neuropharmacol..

[161] Lopez-Sanchez C., Garcia-Martinez V., Poejo J., Garcia-Lopez V., Salazar J., Gutierrez-Merino C. (2020). Early Reactive A1 Astrocytes Induction by the Neurotoxin 3-Nitropropionic Acid in Rat Brain. Int. J. Mol. Sci..

[162] Lopez-Sanchez C., Poejo J., Garcia-Lopez V., Salazar J., Garcia-Martinez V., Gutierrez-Merino C. (2022). Kaempferol Prevents the Activation of Complement C3 Protein and the Generation of Reactive A1 Astrocytes That Mediate Rat Brain Degeneration Induced by 3-Nitropropionic Acid. Food Chem. Toxicol..

[163] Silva Dos Santos J., Cirino J.P.G., de Oliveira Carvalho P., Ortega M.M. (2020). The Pharmacological Action of Kaempferol in Central Nervous System Diseases: A Review. Front. Pharmacol..

[164] Psenicka M.W., Smith B.C., Tinkey R.A., Williams J.L. (2021). Connecting Neuroinflammation and Neurodegeneration in Multiple Sclerosis: Are Oligodendrocyte Precursor Cells a Nexus of Disease?. Front. Cell Neurosci..

[165] Dobson R., Giovannoni G. (2019). Multiple Sclerosis—A Review. Eur. J. Neurol..

[166] Walton C., King R., Rechtman L., Kaye W., Leray E., Marrie R.A., Robertson N., La Rocca N., Uitdehaag B., van der Mei I. (2020). Rising Prevalence of Multiple Sclerosis Worldwide: Insights from the Atlas of Ms, Third Edition. Mult. Scler..

[167] Klineova S., Lublin F.D. (2018). Clinical Course of Multiple Sclerosis. Cold Spring Harb. Perspect. Med..

[168] Ponath G., Ramanan S., Mubarak M., Housley W., Lee S., Sahinkaya F.R., Vortmeyer A., Raine C.S., Pitt D. (2017). Myelin Phagocytosis by Astrocytes after Myelin Damage Promotes Lesion Pathology. Brain.

[169] Mora P., Chapouly C. (2023). Astrogliosis in Multiple Sclerosis and Neuro-Inflammation: What Role for the Notch Pathway?. Front. Immunol..

[170] Kunkl M., Amormino C., Tedeschi V., Fiorillo M.T., Tuosto L. (2022). Astrocytes and Inflammatory T Helper Cells: A Dangerous Liaison in Multiple Sclerosis. Front. Immunol..

[171] Guerrero-Garcia J.J. (2020). The Role of Astrocytes in Multiple Sclerosis Pathogenesis. Neurologia.

[172] Wheeler M.A., Quintana F.J. (2019). Regulation of Astrocyte Functions in Multiple Sclerosis. Cold Spring Harb. Perspect. Med..

[173] Barati S., Kashani I.R., Tahmasebi F. (2022). The Effects of Mesenchymal Stem Cells Transplantation on A1 Neurotoxic Reactive Astrocyte and Demyelination in the Cuprizone Model. J. Mol. Histol..

[174] Sheinerman K.S., Toledo J.B., Tsivinsky V.G., Irwin D., Grossman M., Weintraub D., Hurtig H.I., Chen-Plotkin A., Wolk D.A., McCluskey L.F. (2017). Circulating Brain-Enriched Micrornas as Novel Biomarkers for Detection and Differentiation of Neurodegenerative Diseases. Alzheimers Res. Ther..

[175] Su Y., Chen Z., Du H., Liu R., Wang W., Li H., Ning B. (2019). Silencing Mir-21 Induces Polarization of Astrocytes to the A2 Phenotype and Improves the Formation of Synapses by Targeting Glypican 6 via the Signal Transducer and Activator of Transcription-3 Pathway after Acute Ischemic Spinal Cord Injury. FASEB J..

[176] Moore C.S., Rao V.T., Durafourt B.A., Bedell B.J., Ludwin S.K., Bar-Or A., Antel J.P. (2013). Mir-155 as a Multiple Sclerosis-Relevant Regulator of Myeloid Cell Polarization. Ann. Neurol..

[177] Hess K., Starost L., Kieran N.W., Thomas C., Vincenten M.C.J., Antel J., Martino G., Huitinga I., Healy L., Kuhlmann T. (2020). Lesion Stage-Dependent Causes for Impaired Remyelination in Ms. Acta Neuropathol..

[178] Montagne A., Nikolakopoulou A.M., Zhao Z., Sagare A.P., Si G., Lazic D., Barnes S.R., Daianu M., Ramanathan A., Go A. (2018). Pericyte Degeneration Causes White Matter Dysfunction in the Mouse Central Nervous System. Nat. Med..

[179] Zha Z., Liu Y.J., Liu S.S., Zhang N., Li J.L., Qi F., Jin L.Y., Xue B., Yang T., Fan Y.P. (2022). Bu Shen Yi Sui Capsule Promotes Myelin Repair by Modulating the Transformation of A1/A2 Reactive Astrocytes In Vivo and In Vitro. Oxid. Med. Cell Longev..

[180] Hou B., Zhang Y., Liang P., He Y., Peng B., Liu W., Han S., Yin J., He X. (2020). Inhibition of the Nlrp3-Inflammasome Prevents Cognitive Deficits in Experimental Autoimmune Encephalomyelitis Mice via the Alteration of Astrocyte Phenotype. Cell Death Dis..

[181] Brambilla R. (2019). The Contribution of Astrocytes to the Neuroinflammatory Response in Multiple Sclerosis and Experimental Autoimmune Encephalomyelitis. Acta Neuropathol..

[182] Gorter R.P., Baron W. (2022). Recent Insights into Astrocytes as Therapeutic Targets for Demyelinating Diseases. Curr. Opin. Pharmacol..

[183] Salles D., Samartini R.S., Alves M.T.S., Malinverni A.C.M., Stavale J.N. (2022). Functions of Astrocytes in Multiple Sclerosis: A Review. Mult. Scler. Relat. Disord..

[184] Yadav S.K., Ito N., Soin D., Ito K., Dhib-Jalbut S. (2021). Dimethyl Fumarate Suppresses Demyelination and Axonal Loss through Reduction in Pro-Inflammatory Macrophage-Induced Reactive Astrocytes and Complement C3 Deposition. J. Clin. Med..

[185] De Kleijn K.M.A., Martens G.J.M. (2020). Molecular Effects of Fda-Approved Multiple Sclerosis Drugs on Glial Cells and Neurons of the Central Nervous System. Int. J. Mol. Sci..

[186] Swindell W.R., Bojanowski K., Chaudhuri R.K. (2020). A Novel Fumarate, Isosorbide Di-(Methyl Fumarate) (Idmf), Replicates Astrocyte Transcriptome Responses to Dimethyl Fumarate (Dmf) but Specifically Down-Regulates Genes Linked to a Reactive Phenotype. Biochem. Biophys. Res. Commun..

[187] McGinley M.P., Cohen J.A. (2021). Sphingosine 1-Phosphate Receptor Modulators in Multiple Sclerosis and Other Conditions. Lancet.

[188] Ogasawara A., Takeuchi H., Komiya H., Ogawa Y., Nishimura K., Kubota S., Hashiguchi S., Takahashi K., Kunii M., Tanaka K. (2022). Anti-Inflammatory Effects of Siponimod on Astrocytes. Neurosci. Res..

[189] Colombo E., Bassani C., De Angelis A., Ruffini F., Ottoboni L., Comi G., Martino G., Farina C. (2020). Siponimod (Baf312) Activates Nrf2 While Hampering Nfκb in Human Astrocytes, and Protects from Astrocyte-Induced Neurodegeneration. Front. Immunol..

[190] Trkov Bobnar S., Stenovec M., Mis K., Pirkmajer S., Zore R. (2019). Fingolimod Suppresses the Proinflammatory Status of Interferon-Gamma-Activated Cultured Rat Astrocytes. Mol. Neurobiol..

[191] Du R.W., Bu W.G. (2021). Simvastatin Prevents Neurodegeneration in the Mptp Mouse Model of Parkinson’s Disease via Inhibition of A1 Reactive Astrocytes. Neuroimmunomodulation.

